# Decline of unique Pontocaspian biodiversity in the Black Sea Basin: A review

**DOI:** 10.1002/ece3.8022

**Published:** 2021-09-07

**Authors:** Aleksandre Gogaladze, Mikhail O. Son, Matteo Lattuada, Vitaliy V. Anistratenko, Vitaly L. Syomin, Ana Bianca Pavel, Oana P. Popa, Luis O. Popa, Jan‐Johan ter Poorten, Jacobus C. Biesmeijer, Niels Raes, Thomas Wilke, Arthur F. Sands, Teodora Trichkova, Zdravko K. Hubenov, Maxim V. Vinarski, Olga Yu Anistratenko, Tatiana L. Alexenko, Frank P. Wesselingh

**Affiliations:** ^1^ Naturalis Biodiversity Center Leiden The Netherlands; ^2^ Institute of Environmental Sciences Leiden University Leiden The Netherlands; ^3^ Institute of Marine Biology National Academy of Sciences of Ukraine Odessa Ukraine; ^4^ Department of Animal Ecology & Systematics Justus Liebig University Giessen Giessen Germany; ^5^ Department of Invertebrate Fauna and Systematics Schmalhausen Institute of Zoology National Academy of Sciences of Ukraine Kiev Ukraine; ^6^ Shirshov Institute of Oceanology of Russian Academy of Sciences Moscow Russia; ^7^ Constanta Branch of the National Research and Development Institute for Marine Geology and Geoecology Constanta Romania; ^8^ Grigore Antipa National Museum of Natural History Bucharest Romania; ^9^ Department of Zoology (Invertebrates) Field Museum of Natural History Chicago IL USA; ^10^ Institute of Biodiversity and Ecosystem Research Bulgarian Academy of Sciences Sofia Bulgaria; ^11^ National Museum of Natural History Bulgarian Academy of Sciences Sofia Bulgaria; ^12^ Laboratory of Macroecology and Biogeography of Invertebrates Saint‐Petersburg State University Saint‐Petersburg Russia; ^13^ Department of Cainozoic Deposits Institute of Geological Sciences National Academy of Sciences of Ukraine Kiev Ukraine; ^14^ Kherson Hydrobiological Station National Academy of Sciences of Ukraine Kherson Ukraine; ^15^ Department of Earth Sciences Utrecht University Utrecht The Netherlands

**Keywords:** Black Sea Basin, conservation, human impact, mollusks, Pontocaspian biodiversity, population trends

## Abstract

The unique aquatic Pontocaspian (PC) biota of the Black Sea Basin (BSB) is in decline. The lack of detailed knowledge on the status and trends of species, populations, and communities hampers a thorough risk assessment and precludes effective conservation. This paper reviews PC biodiversity trends in the BSB (Bulgaria, Romania, Moldova, Ukraine, and Russia) using endemic mollusks as a model group. We aim to assess changes in PC habitats, community structure, and species distribution over the past century and to identify direct anthropogenic threats. The presence/absence data of target mollusk species were assembled from literature, reports, and personal observations. Pontocaspian biodiversity trends in the northwestern BSB coastal regions were established by comparing 20th‐ and 21st‐century occurrences. The direct drivers of habitat and biodiversity change were identified and documented. We found that a pronounced decline of PC species and communities is driven by (a) damming of rivers, (b) habitat modifications that disturbed previous natural salinity gradients and settings in the studied area, (c) pollution and eutrophication, (d) invasive alien species, and (e) climate change. Four out of the 10 studied regions, namely, the Danube Delta–Razim Lake system, Dniester Liman, Dnieper–Bug estuary, and Taganrog Bay–Don Delta, contain favorable ecological conditions for PC communities and still host threatened endemic PC mollusk species. Distribution data are incomplete, but the scale of deterioration of PC species and communities is evident from the assembled data, as are major direct threats. Pontocaspian biodiversity in the BSB is profoundly affected by human activities. Standardized observation and collection data as well as precise definition of PC biota and habitats are necessary for targeted conservation actions. This study will help to set the research and policy agenda required to improve data collection to accommodate effective conservation of the unique PC biota.

## Graphical abstract

Distribution data of unique, endemic Pontocaspian (PC) biodiversity of the Black Sea Basin is incomplete, but the scale of deterioration of PC species and communities is evident from the assembled data, as are major direct threats. This review will help to set the research and policy agenda required to improve data collection to accommodate effective conservation of the unique PC biota.

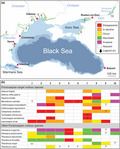

## 1 INTRODUCTION

Pontocaspian (PC) biota forms a unique, endemic ecological community that occurs in transitional brackish habitats between freshwater and marine habitats in the Black Sea region (Anistratenko, 2007b; Mordukhay‐Boltovskoy, 1960; Sowinsky, 1904). Globally, very little endemic biodiversity exists in brackish water systems due to the lack of longevity of these dynamic habitats. Pontocaspian biota evolved in anomalohaline lakes and marginal seas of the Caspian–Black Sea region over the past few million years (Krijgsman et al., 2019; Starobogatov, 1970). Within the Black Sea Basin (BSB) that includes the Azov Sea, PC species live in river deltas, lowland lakes, and estuaries in the northern coastal zones. The current status and trends of PC biodiversity in the BSB are poorly known due to taxonomic uncertainty, lack of standardized observation data, and the transient boundaries of PC habitats (Anistratenko et al., 2020; Sands et al., 2020; Son, 2011a, 2011b, 2011c, 2011d, 2011e, 2011f; Son & Cioboiu, 2011; Wesselingh et al., 2019). This is further hampered by language barriers (e.g., Russia, Ukraine, Romania, Moldova, and Bulgaria all surround the BSB sharing PC habitats and species, yet reporting has mostly been done in their respective languages and often remains unpublished), complex economic situations, and complicated political relationships. While a comprehensive view of the population trends of PC biota is lacking, it is clear that Black Sea coastal areas have faced a variety of anthropogenic modifications. These anthropogenic effects were reported to result in strong reductions in PC species numbers and their abundances in various places (Alexenko & Shevchenko, 2016; Markovsky, 1953, 1954a, 1954b, 1955; Popa et al., 2009; Velde et al., 2019).

**FIGURE 1:**
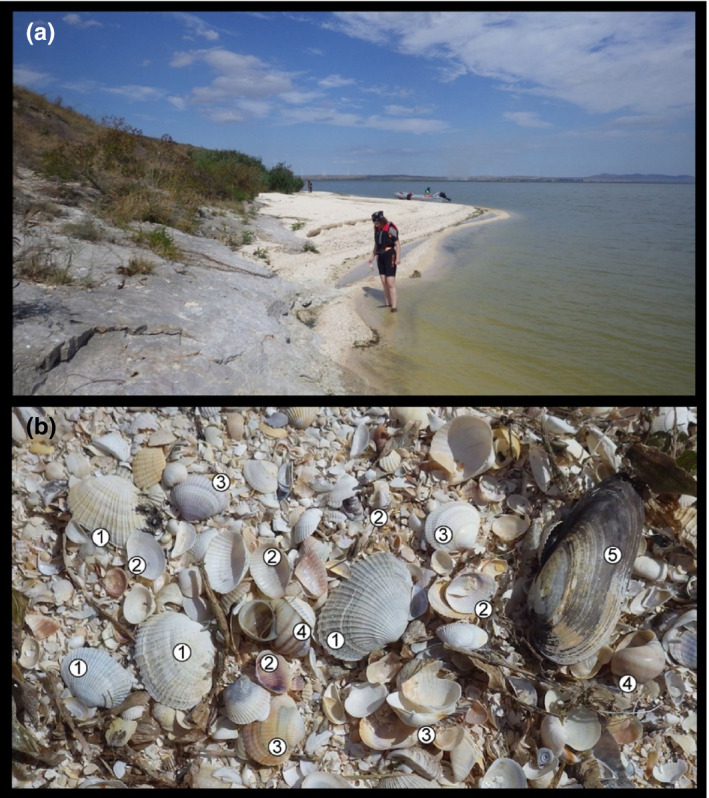
Shells show the decline of PC biota. (a) Shell Beach on Popina Island in northern part of Lake Razim, Romania, located in prime PC habitat (LOP, September 2015). (b) Pontocaspian shell residues showing the extinct *Hypanis plicata* (no. 1), extirpated *Adacna fragilis* (no. 2) and declining *Monodacna colorata* (no. 3). In the past decades, freshwater taxa such as *Viviparus acerosus* (no. 4) and *Unio pictorum* (no. 5) became very abundant while PC species declined. Length of large *Unio* valve is c 8 cm

**FIGURE 2:**
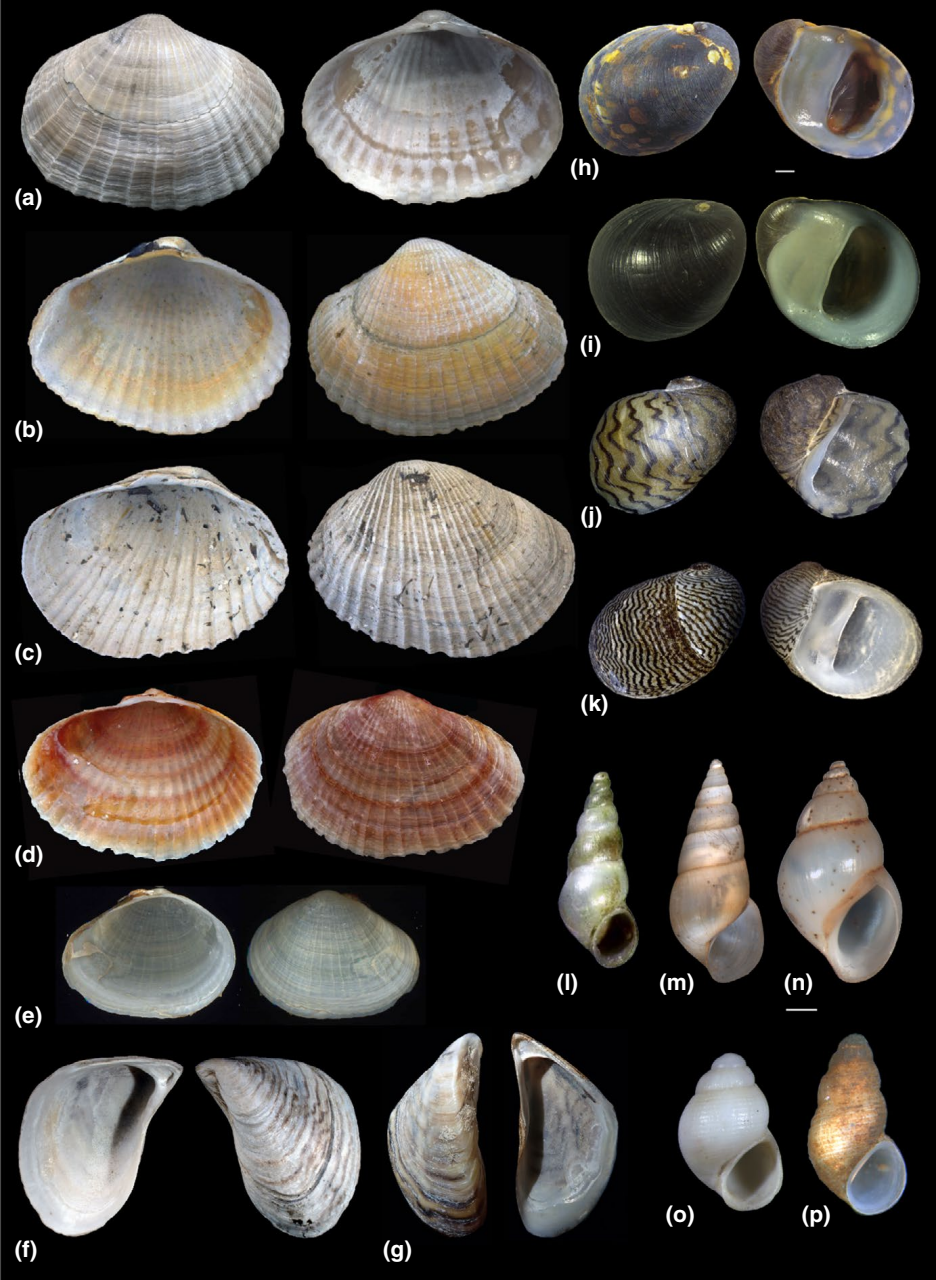
Overview of the PC mollusk species from the northern and northwestern BSB. (a) *Monodacna colorata* (Eichwald, 1829), typical form. Beglitza beach, Taganrog Bay, Azov Sea (Russia). Photo FPW. L 22 mm. (b) *Monodacna colorata* (Eichwald, 1829), forma *pontica*. Lake Razim (Romania). Photo FPW. L 20 mm. (c) *Hypanis plicata* (Eichwald, 1829). Lake Razim (Romania). Photo FPW. L 24 mm. (d) *Adacna fragilis* Milaschewitsch, 1908. Merzhanovo, Taganrog Bay, Azov Sea (Russia). Leg. M. Kurkay, 10.2018, photo JJP. L 17.3 mm. (e) *Adacna vitrea glabra* Ostroumov, 1905. Don River, Tsimlyansk Reservoir (Russia). Photo MOS. L 11 mm. (f) *Dreissena bugensis* Andrussov, 1897. Merzhanovo, Taganrog Bay, Azov Sea (Russia). Photo FPW. L 14 mm. (g) *Dreissena polymorpha* (Pallas, 1771). Southern Bug Liman (Ukraine). Photo MOS. L 21 mm. (h) *Theodoxus fluviatilis* (Linnaeus, 1758) Dnieper River, Kherson Region (Ukraine). Photo VVA. W 8.1 mm. (i) *Theodoxus velox* V. Anistratenko in O. Anistratenko et al. (1999). Dnieper River Delta, Zburjevskiy Liman, Kherson Region (Ukraine). Photo VVA. W 8.4 mm. (j) *Theodoxus danubialis* (Pfeiffer, 1828). Gergweis, Vils River (Germany). Photo AFS. W 10.2 mm. (k) *Theodoxus major* Issel, 1865. Astrakhan, Volga River (Russia). Photo AFS. W 5.5 mm. (l) *Laevicaspia ismailensis* (Golikov & Starobogatov, 1966). Lake Kuhurluy or Yalpuh (Ukraine). Illustration reproduced from Kantor and Sysoev (2006), plate 50, Figure A. L 5.6 mm. (m) *Laevicaspia lincta* (Milaschewitsch, 1908). Lower Dnieper, Kherson (Ukraine). Photo VVA. H 8.97 mm. (n) *Clessiniola variabilis* (Eichwald, 1838). Lower Dnieper, Kherson (Ukraine). Photo VVA. H 7.10 mm. (o) *Clathrocaspia logvinenkoi* (Golikov & Starobogatov, 1966). Lower Don River near Rostov‐on‐Don (Russia). Photo VVA. H 1.58 mm. (p) *Clathrocaspia knipowitschii* (Makarov, 1938). Lower Dnieper, Kherson (Ukraine). Photo VVA. H 1.99 mm

The PC biota comprises vertebrate (e.g., fish), as well as a variety of invertebrate taxa (e.g., mollusks, crustaceans, and worms). Mollusks are particularly well suited to study the changing fate of the PC biota in the BSB (see Son et al., 2020; Velde et al., 2019). They are well represented in museum collections, their shells can indicate previous occurrences of species (Figure 1), they occur in all benthic PC habitats, and several of the species are good environmental indicators (i.e., show sensitivity to oxygen, salinity, water flow, and sedimentation regimes: see Kijashko, 2013; Latypov, 2015; Mordukhay‐Boltovskoy, 1960; Velde et al., 2019; Zhadin, 1952). Within the phylum, some species are characterized by narrow distribution ranges corresponding to narrow ecological tolerance limits. Other species, such as dreissenid bivalves, are opportunistic and have become major invaders elsewhere (Orlova et al., 2005). The taxonomic status of several PC mollusk species is not resolved due to large morphological variability (see Figure 2a,b) and is hampered by the paucity or absence of living material for novel DNA‐based research (Wesselingh et al., 2019). However, a network of PC mollusk specialists has been established in the past years as part of the European Union funded Innovative Training Network “PRIDE” (www.pontocaspian.eu) that is actively targeting taxonomic uncertainties, which is an ongoing effort and provides an essential taxonomic base for this study.

This paper aims to review distribution trends of PC biota (using mollusks as a model group) in the BSB by comparing historical (20th century) and modern (21st century) occurrences. Furthermore, we aim to identify the direct anthropogenic threats to their existence and survival (sensu Díaz et al., 2015), viz., processes and settings resulting from human decisions and actions that have direct implications for turnover/decline of PC biota, such as uncontrolled influx of sewage, invasion of alien species, and establishment of large dammed reservoirs in river basins (Shiganova, 2011, Semenchenko et al., 2015, Lattuada et al., 2019, e.g., Lattuada et al., 2020). Pontocaspian biodiversity is also affected by indirect anthropogenic drivers such as the organization and interaction within and between societies, stakeholders, and people and their interactions with nature. For the BSB, these are treated elsewhere (Gogaladze, Raes, et al., 2020; Gogaladze, Wesselingh, et al., 2020). Based on this review, we outline follow‐up approaches to develop a conservation strategy that applies to the entire PC benthic biota in the BSB.

## 2 BACKGROUND

### 2.1 Pontocaspian mollusk species in the Black Sea Basin

Most of the PC species evolved from ancestral species that radiated in the Late Miocene and Pliocene Paratethyan Basins (Krijgsman et al., 2019). The common historical origin of PC species and related ecological adaptations distinguishes this group from other groups such as Palearctic freshwater species groups and several opportunistic marine species occurring in the PC region today (Anistratenko, 2007b; Sowinsky, 1904; Starobogatov, 1970; Wesselingh et al., 2019; Zhadin, 1952).

The historical distribution of PC mollusk families in the BSB has been the subject of various studies, for example, Hydrobiidae (Alexenko & Starobogatov, 1987; Anistratenko, 2007a, 2007b, 2008; Golikov & Starobogatov, 1966, 1972; Grossu, 1962; Makarov, 1938; Sitnikova & Starobogatov, 1999; Wilke et al., 2007), Neritidae (Anistratenko et al., 1999, 2011, 2017, 2020; Golikov & Starobogatov, 1966, 1972; Lindholm, 1908; Makarov, 1938; Mordukhay‐Boltovskoy, 1960; Sands et al., 2020), Lymnocardiinae (Anistratenko et al., 2011; Borcea, 1926a, 1926b; Grossu, 1973; Makarov, 1938; Milaschewitsch, 1916; Munasypova‐Motyash, 2006; Ostroumov, 1898; Popa et al., 2009), and Dreissenidae (Andrussov, 1897; Rosenberg & Ludyanskiy, 1994; Son, 2007b).

### 2.2 Habitats of Pontocaspian species and communities in the Black Sea Basin

**FIGURE 3:**
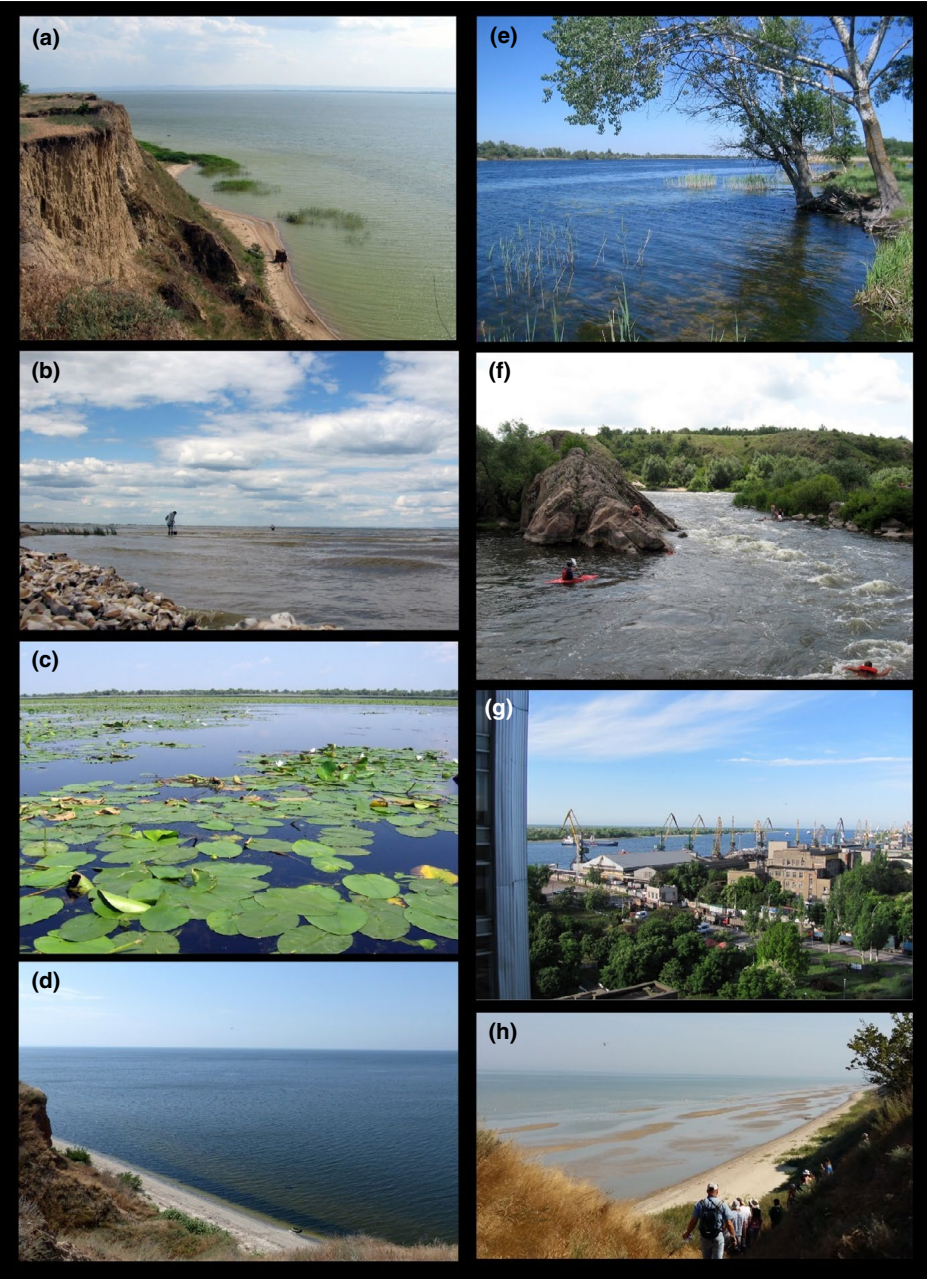
Examples of PC habitats in the BSB. (a) Lake Yalpuh, Ukraine (MOS, June 2009). This large lake is still a prime PC habitat; however, eutrophication is noticeable. The reed vegetation zone along the shore is a habitat for PC hydrobiid species. (b) Dniester Liman, Ukraine (VVA, June 2016). The small, waves are actively forming shell ridges along the liman near Belgorod––Dnestrovsky that are mainly composed of *Monodacna* and *Dreissena* shells. *Theodoxus* and mostly juvenile *Monodacna* are still living in the area, and hydrobiids are represented by fresh empty shells. (c) Lake Beloye in Dniester Delta, Ukraine (photo MOS, July 2009). Smaller deltaic lakes and river floodplain lakes, such as shown in this image, hosted a combination of freshwater and PC species in the past (< 20th century), but PC species have mostly disappeared from these habitats in the past century. (d) Dnieper Liman, Aleksandrovka, Ukraine (VVA, June 2016). Sandy bottom of the distal sector of the liman. Freshwater species are dominant here. Large quantities of empty shells of PC species such as hydrobiid, *Theodoxus,* and *Monodacna* spp. are indicative of their former abundance in the region. (e) Dnieper Delta, Konka Branch (MOS, May 2007). Wide riverine channel upstream the estuary. All groups of PC mollusks are present in this habitat. (f) Rapids of the Southern Bug River, Migia Canyon, Ukraine (MOS, July 2009). These rapids form a natural upper boundary for the distribution of most PC taxa. (g) Kherson cargo harbor, Ukraine (VVA, May 2016). The harbors are important vectors for invasive species, and the dredging required to ensure access to sea has various impacts on PC habitats in the estuaries and limans. (h) Taganrog Bay at Semibalki, Russia (FPW, September 2017). The view shows the shallow nature of the bay and the sandy character of the sediments. Here, large populations of *M*. *colorata* and *A*. *fragilis* occur

**FIGURE 4:**
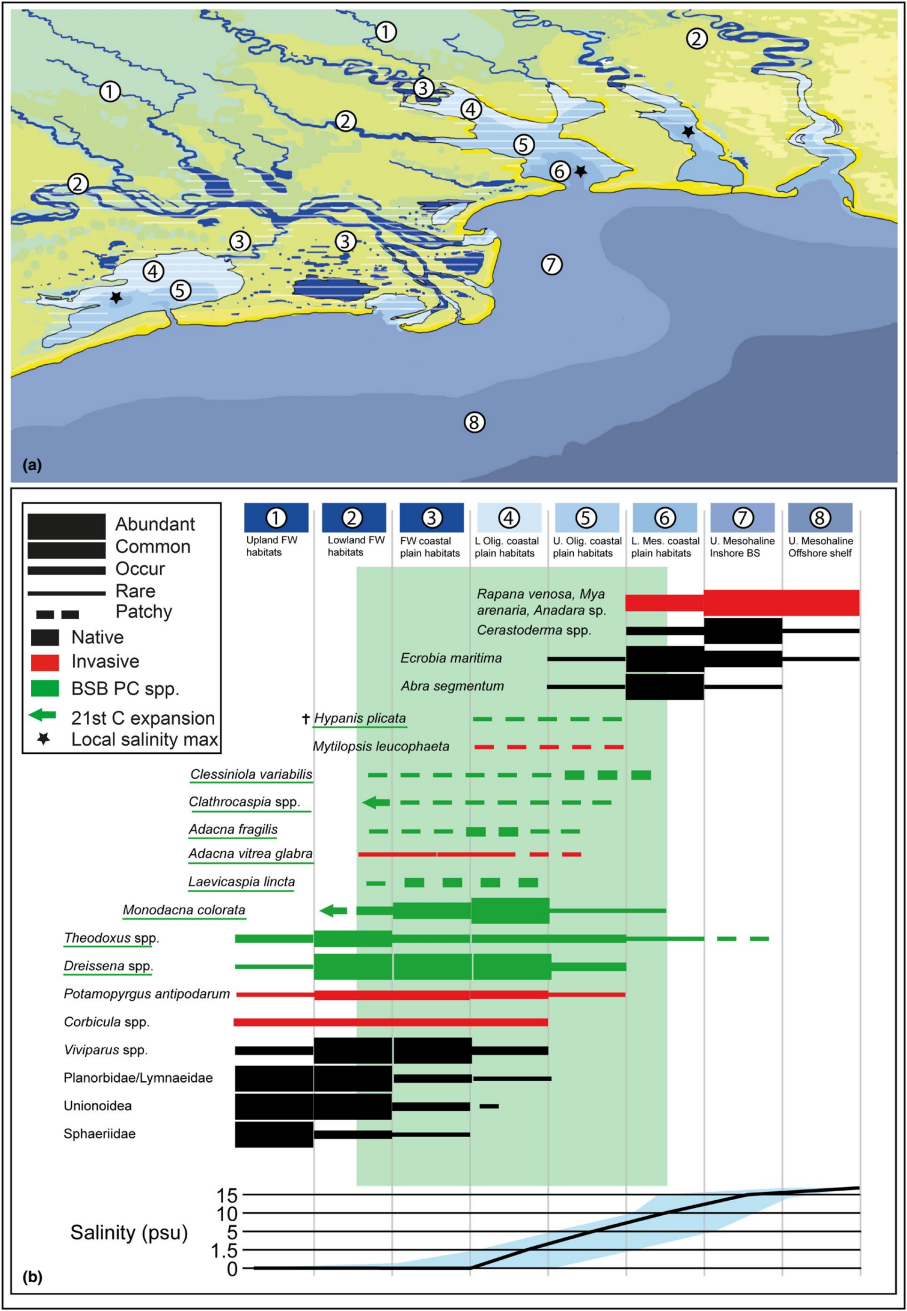
Simplified model of coastal landscapes depicting habitats of selected PC (green underlined) and other abundant mollusk species in the northwestern Black Sea coastal zone for the 20th‐21st century. The optimum PC habitats are shaded (above) and indicated in green (below). FW—freshwater, U—Upper, L—Lower, Olig—Oligohaline, Mes—Mesohaline. Our model summarized personal observations as well as published accounts. In each sub‐basin in the BSB, the salinity gradients and habitat successions are complex. In some areas, local salinity maxima occur that are the result of excessive evaporation rather than a simple freshwater to marine gradient

Pontocaspian communities occur(ed) in coastal plains in areas influenced by the Black Sea and Azov Sea, such as lower stretches of rivers, lagoons, delta areas, estuaries/limans, and bays (Figures 3 and 4). Limans (a particular landform common to the North Black Sea) are estuaries or lagoons mostly or entirely separated from the sea by sand barrier systems and have lagoonal, lake, bay, and estuarine properties. Some PC groups, such as *Theodoxus* and *Dreissena* species, are tolerant to a wide array of environmental conditions and have far larger distribution ranges than lymnocardiine and/or hydrobiid species—they are abundant in rivers and lakes, including those outside the BSB drainage systems (Sands et al., 2020; Zhadin, 1952).

Three main PC community types have been described during the 20th century from the different regions: (1) *Dreissena* communities, (2) *Dreissena*–*Monodacna* communities, and (3) *Adacna*–*Hypanis–Monodacna* communities. *Dreissena*‐dominated communities are common in rivers (often with the presence of *Theodoxus* spp.) within and outside the PC region, but also occur as secondary, species‐depleted communities in estuaries in all BSB PC regions (Markovsky, 1953, 1954a, 1955; Mordukhay‐Boltovskoy, 1960; Zhadin, 1931). Several *Dreissena* subcommunities have been proposed, and all are characterized by the absence of *Monodacna*. The *Dreissena*–*Monodacna* communities form species‐rich communities in freshwater to oligohaline settings at the core of estuaries in all BSB PC regions and are locally dominated by either *Monodacna* or *Dreissena* species (Markovsky, 1953, 1954a, 1955; Mordukhay‐Boltovskoy, 1960). *Adacna*–*Hypanis–Monodacna*‐dominated communities were common in the oligohaline–mesohaline zones in all BSB PC regions (Markovsky, 1953, 1954a, 1955; Mordukhay‐Boltovskoy, 1960; Shokhin et al., 2006; Zhadin, 1931). These communities were relatively species‐poor, containing only *Adacna fragilis*, *Monodacna colorata,* and *Hypanis plicata*. However, with the demise of the latter in the BSB, these communities vanished. Within the central‐eastern parts of the Taganrog Bay today an impoverished version of the community exists (lacking *Hypanis*) that is often termed *Monodacna* community (Nekrasova, 1972; Stark, 1960; Vorobyev, 1949). The optimum conditions for this community are fresh to oligohaline waters (up to 5 psu), sandy, shelly, or moderately silty substrate in the bay and low current areas, which are indicative of good oxygenation and moderate hydrodynamics (such as habitats in the outer Don River). Within the PC habitats previously local very dense aggregates of PC gastropod occurrences existed that may be interpreted as communities or subcommunities. *Clessiniola variabilis*‐dominated communities have been mentioned from shallow waters with variable salinities in the Dniester and Dnieper–Bug regions (Markovsky, 1953, 1954a), but we have not encountered such aggregates in the past decades. *Laevicaspia lincta*‐dominated communities (mentioned from Dniester and Kuchurgan limans, Katlabukh, Yalpuh, and Dnieper by Markovsky, 1953, Markovsky, 1954a, Markovsky, 1955, Olivari, 1953, and observed in Razim Lake by Wilke et al., 2007 as late as in 2003) were a common feature in freshwater areas and occasionally low oligohaline water settings with abundant *Dreissena*.

## 3 METHODS

In February 2020, the authors’ team assembled scientific papers, reports, and secondary literature available in English, Russian, Romanian, Bulgarian, and Ukrainian to document the occurrence and trends of target PC mollusk species in coastal regions of the northeastern BSB since the 20th century. Selection of the literature and reports was based on personal knowledge and extensive research experience of the authors’ team in the BSB. Identified literature was then reviewed, and additional relevant articles were identified through the reference lists of the papers and reports, a method referred to as the “backward snowballing” (Jalali & Wohlin, 2012; Kitchenham & Charters, 2007). We combined the retrieved presence/absence data of target mollusk species from literature and reports with the personal observations of authors. Species trends of target PC mollusks in the northwestern BSB coastal regions were established by comparing 20th ‐ and 21st‐century occurrences. Additionally, direct drivers of habitat and biodiversity change were identified in publications and reports and documented.

We defined Pontocaspian (PC) mollusk species as extant, endemic, fully aquatic species, which evolved in the Black Sea and Caspian Sea Basins during the Quaternary, where they became adapted to a range of anomalohaline salinity regimes that characterized these basins. We based this review on endemic and native PC mollusk species (Figure 2, Table 1) that have been reported alive from BSB coastal habitats in the 20th and 21st centuries (following the taxonomy of Wesselingh et al., 2019, and Sands et al., 2020 and with taxonomical updates: see Appendix S1).

**TABLE 1:** Taxonomic status of PC mollusk species from the Black Sea Basin (BSB) with confirmed living 20th‐ and 21st‐century occurrences. ^1^Wesselingh et al. (2019); ^2^Sands et al. (2020); ^3^Son et al. (2020); ^4^Appendix S1

(Sub) Family	Species	Author	Status
Lymnocardiinae	*Adacna fragilis*	Milaschewitsch (1908)	BSB endemic^4^
Lymnocardiinae	*Adacna vitrea glabra*	Ostroumov (1905)	Caspian invasive^3,4^
Lymnocardiinae	*Hypanis plicata*	Eichwald (1829)	PC endemic^1^
Lymnocardiinae	*Monodacna colorata*	Eichwald (1829)	BSB endemic (20th century), now invasive in Caspian basin
Dreissenidae	*Dreissena bugensis*	Andrussov (1897)	BSB endemic (<20th century), now global invasive
Dreissenidae	*Dreissena polymorpha*	Pallas (1771)	Native^1^
Neritidae	*Theodoxus danubialis*	Pfeiffer (1828)	Native^1,2^
Neritidae	*Theodoxus fluviatilis*	Linnaeus (1758)	Native^1,2^
Neritidae	*Theodoxus major*	Issel (1865)	PC native^2^
Neritidae	*Theodoxus velox*	V. Anistratenko in O. Anistratenko et al. (1999)	PC native^2^
Hydrobiidae	*Clathrocaspia knipowitschii*	Makarov (1938)	BSB endemic (20th century), now possibly invasive in Danube catchment^1^
Hydrobiidae	*Clathrocaspia logvinenkoi*	Golikov and Starobogatov (1966)	BSB endemic^1^
Hydrobiidae	*Clessiniola variabilis*	Eichwald (1838)	PC endemic^1^
Hydrobiidae	*Laevicaspia lincta*	Milaschewitsch (1908)	BSB endemic^1^
Hydrobiidae	*Laevicaspia ismailensis*	Golikov and Starobogatov (1966)	BSB endemic^1^
Hydrobiidae	*Turricaspia chersonica*	Alexenko and Starobogatov (1987)	BSB endemic

We defined optimum PC habitats as waterbodies (e.g., lakes, estuaries, bays, and river stretches) where at least one endemic PC species of two different families co‐occur (Table 1). Our definition will need expansion when other groups in addition to mollusks are included. Optimum PC habitats contain(ed) communities dominated by PC species within the coastal zone, mostly in oligohaline settings (Alexenko & Starobogatov, 1987; Anistratenko, 2007b; Anistratenko et al., 2011; Makarov, 1938; Munasypova‐Motyash, 2006; Starobogatov, 1970; Zhadin, 1952), where the densities of PC mollusks are variable. *Dreissena* and *Monodacna* can dominate communities, but most of the PC hydrobiids have patchy occurrences (Alexenko & Kucheryava, 2019; Alexenko & Starobogatov, 1987; Anistratenko & Anistratenko, 2018).

### 3.1 Pontocaspian habitat mapping

We retrieved freshwater habitat polygons from the HydroLAKES dataset (https://www.hydrosheds.org/pages/hydrolakes) to map the PC habitats in the BSB using QGIS 3.10 “A Coruña.” We manually edited those polygons that did not cover the PC habitats, such as swamps and marshes, based on published literature and expert knowledge. We also manually drew lagoons and bays of Pontocaspian habitats, which are not part of the HydroLAKES dataset based on published accounts and expert knowledge. Given the densely aggregated small lakes in the Danube Delta with surface areas lesser than 0.2 km^2^, we merged the Chilia branch of the Danube River and outer delta lakes both upstream and downstream of Vilkovo (Table A2.1 and Appendix S3).

## 4 RESULTS

### 4.1 Status and trends of Pontocaspian species in the Black Sea Basin

**FIGURE 5:**
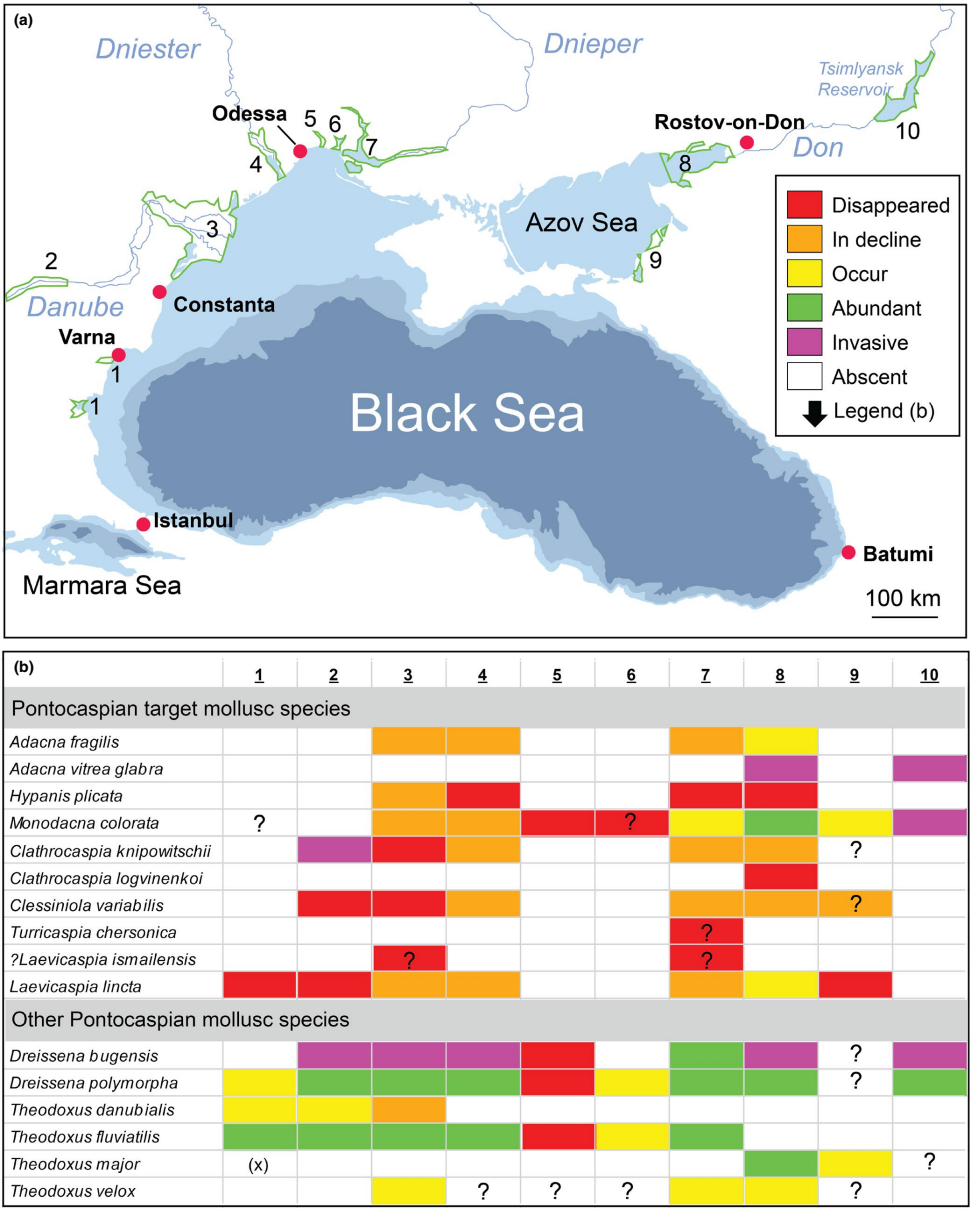
(a) PC species occurrences in the BSB. 1. Bulgarian coastal lagoons and limans, 2. Lower Danube River, 3. Danube Delta–Razim, 4. Dniester Liman, 5. Tiligul Liman, 6. Berezan Liman, 7. Dnieper–Bug Estuary, 8. Taganrog Bay–Don Delta, 9. SE Azov Sea coast, 10. Tsimlyansk Reservoir. (b) Status of PC mollusk species. “Decline” stands for diminished distribution range within an area and/or declining abundances in the past century. “Invasive” stands for 21st‐century introductions. Question marks denote areas with insufficient observations (such as southeast Azov coast) or taxonomic groups that require re‐examination (*Theodoxus* species). *Earlier reports of this species likely to be misidentifications of *T*. *fluviatilis* and/or *T. danubialis* (AFS, PO)

Status and trends of PC mollusk species are based on data derived from 68 published accounts and personal observations (PO) of the authors (ABP, AFS, FPW, LOP, MOS, OPP, TW, MVV, VVA, OYA, VLS, and TT). Compiled data were mostly qualitative resulting in unspecified number of records. Ten regions in the BSB contain 20th‐ and/or 21st‐century occurrences of endemic PC species (Figure 5). Historical (20th century) and modern (21st century) distributions of PC target taxa are summarized in Appendix S2. PC habitat polygon shapefiles and the attributes describing historical (20th century) and modern (21st century) distributions of PC target taxa are provided in Appendix S3.

#### 4.1.1 Bulgarian coastal lagoons and limans

The Bulgarian Black Sea coast contains 31 wetland areas such as lakes, marshes, and lower river floodplain areas (Varbanov, 2002), from where living PC species and shells have been reported (Georgiev & Hubenov, 2013; Hubenov, 2007, 2015; Sands et al., 2019; Appendix S2). *Theodoxus fluviatilis* has been reported from more than 15 wetlands (Hubenov, 2015), while *Dreissena polymorpha* occurred in about ten wetlands in the past, and currently is confirmed from five of these native habitats (Hubenov, 2015; Vidinova et al., 2016). *Theodoxus danubialis* (reported as *T. pallasi*) occurred in Lake Varna before salinization in the first half of the 20th century (Drensky, 1947; Kaneva‐Abadjieva, 1957; Sands et al., 2020) and is now considered extinct in Bulgaria (Hubenov, 2015). Living specimens of *L*. *lincta* (reported as *Micromelania lincta*) were recorded in Lake Mandra (June 1944) and Lake Beloslav (August 1945) by Drensky (1947). The species was considered rare for Bulgaria (Drensky, 1947), and since then, no further occurrences have been recorded (Hubenov, 2015). Pontocaspian cardiids have been reported only as shells in the Bulgarian coastal wetlands. Kaneva‐Abadjieva (1957) found single shells of *M*. *colorata* at different parts and depths of Lake Varna, assuming that the species was present there before salinity regime change in the first half of the 20th century. Shells of *L. lincta*, *M. colorata,* and *H*. *plicata* (reported as *Adacna relicta* and *A. plicata relicta*) have been reported from the Black Sea littoral sediments by Valkanov (1957), Marinov (1990), and Hubenov (2015) and shells of *C. variabilis* reported by Genov and Peychev (2001) and Hubenov (2015). It is unclear whether these littoral shells represent possible 20th‐century occurrences, as older Holocene and even Late Pleistocene occurrences are well known from shallow deposits in the Black Sea coastal and shelf areas (Velde et al., 2019).

The Bulgarian Black Sea coastal wetlands have been exposed to a variety of strong anthropogenic pressures owing to agricultural, recreational, urban, and industrial development over the past two centuries (Hubenov, 2015; Trichkova, 2007). Increased eutrophication and substantial variation in physico‐chemical parameters such as salinity, oxygen content, mineral content, and temperature in the wetlands have caused pronounced changes in benthic invertebrate communities (Trichkova, 2007). Some of the past habitats sustaining PC species have completely changed. For example, Lake Varna was connected to the sea through a navigation canal in 1909 and to Lake Beloslav in 1923. Later, in 1975, a bigger canal and a sea port were built, increasing salinity within both lakes, driving the loss of their natural fauna, including PC species (Trichkova, 2007; Varbanov, 2002). Benthic invertebrate biota in other wetlands (e.g., Durankulak, Shabla‐Ezerets, Burgas, Mandra, and Dyavolsko Blato Marsh) declined or vanished due to restriction or complete disconnection from the Black Sea because of damming, and/or due to intensive fish‐farming activities, overfishing, and household and industrial pollution (and Trichkova, 2007, summarized in Hubenov, 2015).

#### 4.1.2 Lower Danube River

*Theodoxus* and *Dreissena* are and have always been common in the Danube River (Angelov, 2000; Russev, 1966; Sands et al., 2019; Trichkova et al., 2019). In the Bulgarian sector, PC hydrobiid shells were reported in the 20th century. In June 1958, empty shells of *L*. *lincta* (reported as *M*. *lincta*) were recorded at Oryahovo (678 rkm) by Russev (1966). Shells of *C*. *variabilis* were found upstream of Lom (474 rkm) in September 1957, at Ruse (493 rkm) in October 1959, and upstream of Silistra (381 rkm) in June 1963 (Russev, 1966). No 21st‐century records exist of these PC hydrobiids from the Bulgarian Danube River stretch. However, recently a *Clathrocaspia* sp. has been described as *Caspia milae* in Boeters et al. (2015) from Vardim Island in the Bulgarian sector of the Danube, whose identity is subject to further study (see Appendix S1).

The main threats to the aquatic mollusks in general and the PC fauna in the Lower Danube River in particular are the loss and degradation of habitats, pollution, and introduction of invasive alien species (Trichkova et al., 2019). Throughout the years, the Danube River has been contaminated by urban, industrial, and agricultural waste and has experienced increasing economic activities, such as ship traffic (Russev & Naidenow, 1978). A major threat that has become a problem in the 21st century is the introduction, establishment, and spread of invasive alien species (Paunović & Csányi, 2018). In recent years, owing to the increase in abundance and biomass of the newly introduced invasive alien mussels *Corbicula fluminea*, *Sinanodonta woodiana*, and *Dreissena bugensis*, benthic habitats in the Bulgarian sector of the Danube River completely changed (Hubenov, 2001, 2006; Hubenov & Trichkova, 2007; Hubenov et al., 2012, 2013), which may have potential adverse impacts on several PC species. Additionally, the invasive mussels may directly impact PC species through competition and fouling.

#### 4.1.3 Danube Delta–Razim Lake system

**FIGURE 6:**
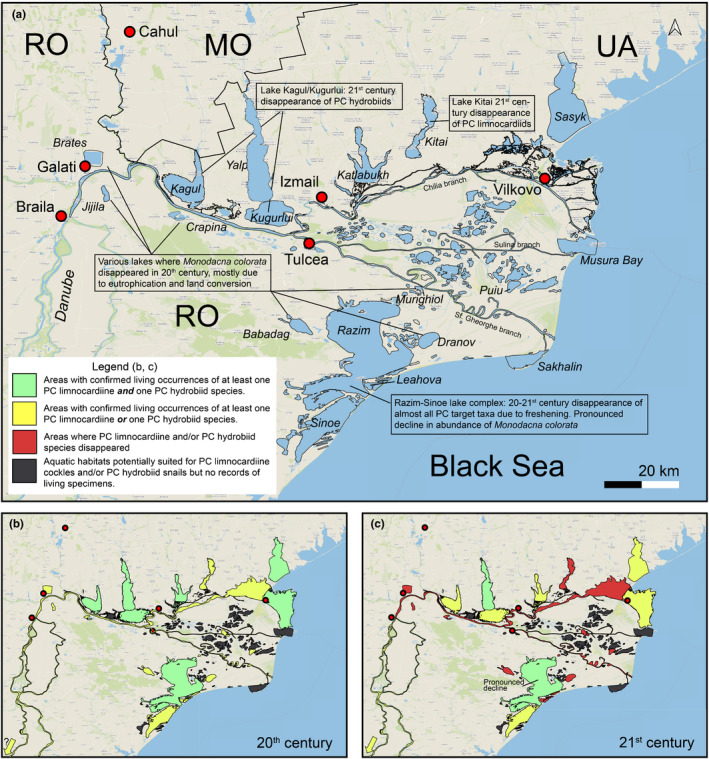
Pontocaspian habitats in the Danube Delta region. (a) Regional overview and major trends, (b) 20th‐century occurrences, (c) 21st‐century occurrences. See data in Appendix S2, Table A2.1, outline of subareas in Figure A2.1. Pontocaspian taxa still appear in Razim Lake complex in 21st century (hence the green color), but hydrobiid species have not been reported after 2003 and lymnocardiine species have strongly declined in abundance (*M*. *colorata*) or disappeared (*Adacna* and *Hypanis* spp.). Map is projected in EPSG Projection 4,326—WGS 84

The Danube Delta (up to its apex near Galati), the neighboring drowned valley lakes both on the Romanian side (e.g., Brates, Crapina, and Jijila) and on the Ukrainian side (Yalpuh, Katlabukh, Kagul, and Kitai), and the coastal Razim–Sinoe Lake complex to the south of the delta and Sasyk Lake to the north make up a large (c 6,000 km^2^) and varied area that hosts many PC species (Figure 6). Lake Sasyk was historically separated from the Danube Delta, but was included when, in 1978, a feeder channel from the Danube was constructed. Most of the Danube–Razim region consists of freshwater habitats (e.g., river channels, floodplain delta lakes, drowned river valleys, and swamps) but, importantly, salinity gradients toward mesohaline settings occur in the outer delta and in the coastal lagoons and lakes. The maximum depth within the Razim Lagoon complex is 3.5 m (Velde et al., 2019).

The Danube Delta region historically harbors a diverse PC mollusk fauna (Markovsky, 1955; Mordukhay‐Boltovskoy, 1960; Popa et al., 2009; Velde et al., 2019) with twelve PC species (Figure 6). Common PC mollusk species are *M*. *colorata*, *T*. *fluviatilis*, and *D*. *polymorpha*. All three lymnocardiine species recorded in the 20th century have disappeared in Romanian lakes, with the exception of the Razim–Sinoe (Popa et al., 2009; Velde et al., 2019), where *M. colorata* and *A*. *fragilis* have still been recorded in the 21st century. However, annual fieldwork in the Razim complex has shown that their abundance has strongly declined in the past 15 years (Popa et al., 2009). In the 20th century, *H. plicata* was common in the Razim–Sinoe Lake complex (Teodorescu‐Leonte, 1966). The last time this species was found alive in Razim–Sinoe Lake complex was in 2004 (Tatiana Begun, PO). Within the lakes and lagoons very close to the Black Sea coast, *A. fragilis* has been a common occurrence in the 20th century (Borcea, 1926b; Grossu, 1962; Markovsky, 1955), but the species has declined recently (Popa et al., 2009). Velde et al. (2019) showed that the Razim communities have almost entirely been replaced by freshwater communities in the past decades. In Romania, PC hydrobiid species were reported mostly from the Razim–Sinoe complex and low salinity habitats near the mouth of the Danube distributaries (Grossu, 1956). In most cases, these records are represented by empty shells and their historical distribution (e.g., 20th‐century occurrences) is not well known. In the past decade, no living specimens were encountered apart from a 2003 record of *L. lincta* (Wilke et al., 2007).

In the Ukrainian part of the Danube Delta, in the Kitai Lake, PC communities have recently disappeared completely and PC species abundances in this lake and in other lakes are decreasing (MOS and VVA, PO). The distribution ranges of *L*. *lincta* and *A*. *fragilis* have decreased compared with occurrences reported over a century ago (Markovsky, 1953, 1954a, 1954b, 1955; Milaschewitsch, 1916; Ostroumov, 1898). The latter species became rare in its native NW Black Sea coastal range (Lyashenko et al., 2012; Munasypova‐Motyash, 2006), but became temporarily abundant (along with *M*. *colorata*) in Lake Sasyk when the lake was connected to the Danube River, via a canal, in 1978 (Khalaim & Son, 2016). Previously, Lake Sasyk hosted marine communities, but after the connection with the Danube River was established, two PC communities became common there, viz., *Dreissena* communities in the shore zones and *Monodacna* communities in deeper parts. *Laevicaspia ismailensis* may have disappeared from lakes Yalpuh and Kuhurluy (VVA, MOS, PO).

Several causes have been proposed for the decline of PC species and communities in the Danube–Razim region. Eutrophication and conversion of inland lakes were linked by Popa et al. (2009) to the disappearance of lymnocardiine species. Velde et al. (2019) related the breakdown of the salinity gradients in the Razim–Sinoe Lake complex, due to rerouting of Danube waters as well as closing Black Sea inlets in the second half of the 20th century, to the collapse of PC communities and disappearance of species. Recently, invasive *Corbicula* spp. have been expanding in the Danube Delta area (Pavel et al., 2017) and potential interactions of this successful invasive (Crespo et al., 2015) with PC species are a reason for concern.

#### 4.1.4 Dniester Liman

**FIGURE 7:**
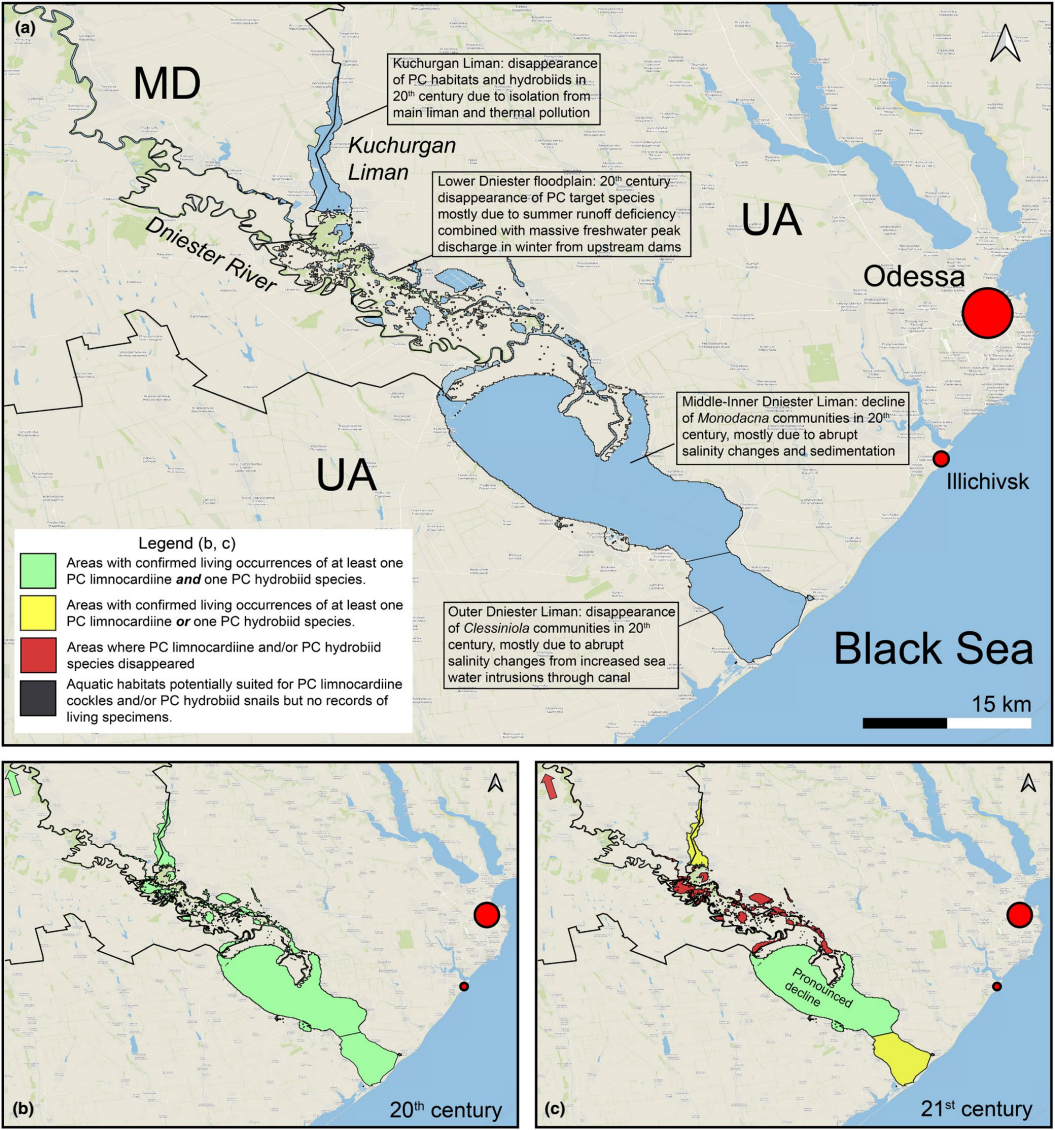
Pontocaspian habitats and trends in the Dniester Liman. (a) Regional overview and major trends, (b) 20th‐century occurrences, (c) 21st‐century occurrences. See data in Appendix S2, Table A2.2, outline of subareas in Figure A2.2. Map is projected in EPSG Projection 4,326—WGS 84

The lower Dniester, comprising the Dniester Delta and Liman, the Kuchurgan Liman (Figure 7), and the lower Dniester River up to Dubăsari Dam (Moldova) historically host a rich array of PC fauna that includes 10 mollusk species (Grinbart, 1953a; Markovsky, 1953; Son, 2007b). The Dniester Liman is about 45 km long, with a surface area of about 400 km^2^, and a maximum depth is 2.7 m. In the 20th century, the Liman was subdivided into an inner freshwater‐oligohaline zone (up to 0.5 psu), a middle oligohaline zone (up to 4 psu), and an outer mesohaline zone (salinities typically between 4 and 9 psu with episodic lowering during peak floods; Markovsky, 1953). Salinity regimes changed due to human interference. A deep‐water sea canal has enabled seawater intrusions during storm surges. In the upper Dniester basin, a system of fish ladders decimated natural flow regimes (Zhulidov et al., 2015). In general, the lower Dniester basin is characterized by problems of seasonal runoff deficiency and associated degradation of floodplain ecosystems, common to all large PC rivers with cascades of dams (Shevtsova, 2000). The episodic release of large amounts of freshwater from reservoirs in the feeding rivers causes strong episodic freshening of the inner and middle parts of the Dniester system. This freshening sharply steepens the salinity gradient and minimizes optimum salinity areas of PC biota. The Kuchurgan Liman (a part of the Dniester Liman that became cut off by the prograding river delta) was turned into a cooling pond for the power station and has thus become impacted by thermal pollution.

The distribution range of PC communities in the Dniester Delta declined in the early 20th century before the start of large‐scale anthropogenic modifications, such as the construction of dams and canals and thermal pollution (Grinbart, 1953a; Markovsky, 1953). According to our observations (MOS, VVA), PC lymnocardiine and hydrobiid species have completely disappeared in floodplain lakes and only the most tolerant *Dreissena* and *Theodoxus* species have survived in river channels. In the past decades, the Dniester Liman communities dominated by *A*. *fragilis* and *H*. *plicata* have vanished. On species level, *A*. *fragilis*, *M. colorata,* and *L*. *lincta* have a considerably reduced distribution ranges and/or abundances and *H*. *plicata* and *Clathocaspia knipowitchii* are possibly extinct in the Dniester area (VVA, PO).

Dam construction has been a major driver for Dniester floodplain ecosystem demise (Shevtsova, 2000), which has been further affected by an increase in water extraction, climate change, and organic pollution. Increased episodic intrusions of seawater and variability of freshwater inflow from the catchments have severely impacted the salinity gradients. Salinity increase in estuaries under the conditions of climate change and artificial flood‐changing constructions is a global trend (Rahel & Olden, 2008). In freshwater and oligohaline zones, among numerous alien species, two species of mollusks (a) *D*. *bugensis*, a PC species from the Dnieper–Bug Estuary, and (b) *Potamopyrgus antipodarum*, a species from New Zealand, have affected the original PC communities (Son, 2007a, 2008). In the lower zone of the Dniester Liman, alien species (especially *Mytilopsis leucophaeta*) occupy the vacant niches of PC species, which are not adapted to rapid salinity changes (Zhulidov et al., 2015). These invasive species, in the lower zone of the Dniester Liman, have taken advantage of the PC species decline, but have not necessarily been demonstrated to have driven the reduction and disappearance of PC communities.

#### 4.1.5 Tiligul Liman

**FIGURE 8:**
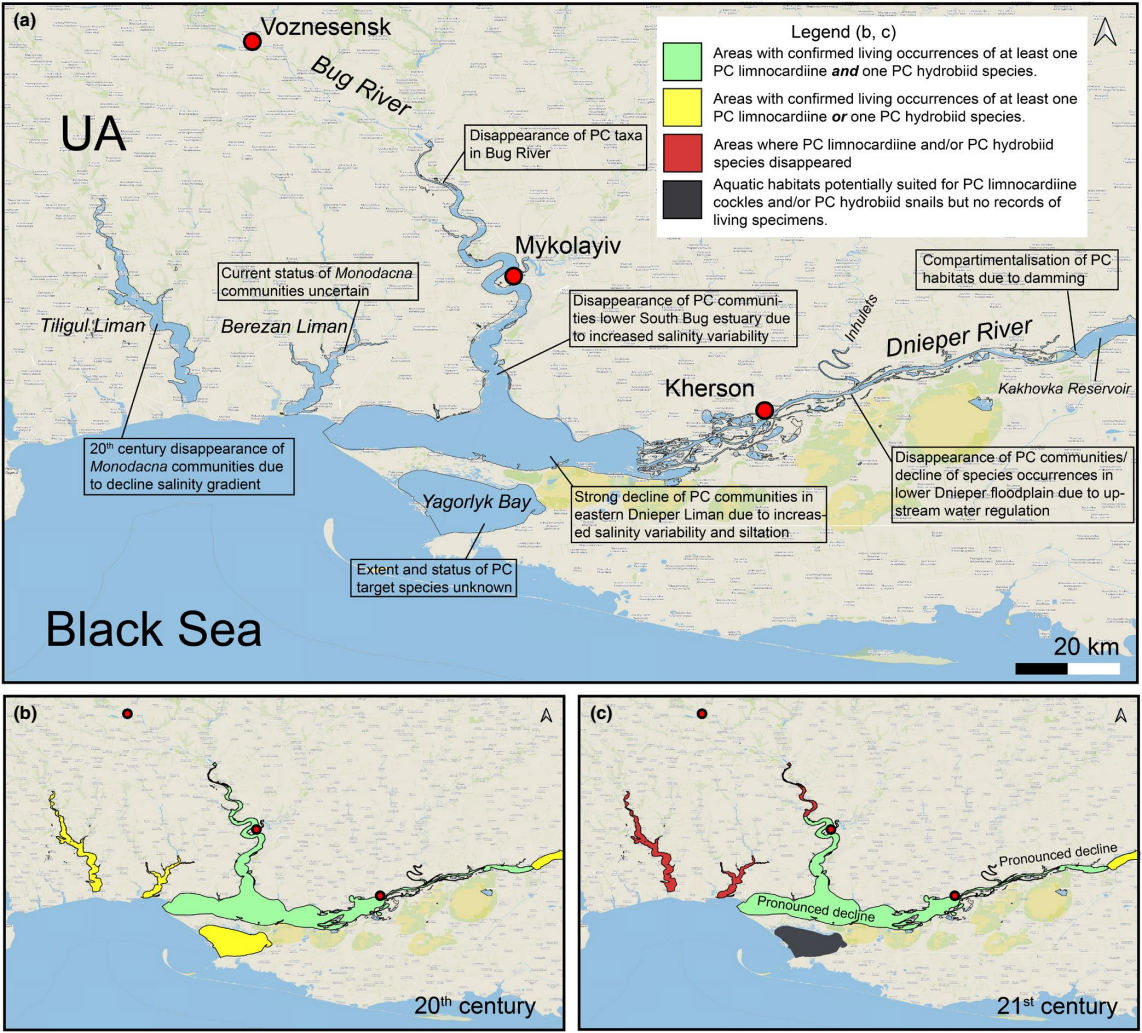
Pontocaspian habitats and trends in the Dnieper–Bug Estuary and adjacent Tiligul and Berezan Limans. (a) Regional overview and major trends, (b) 20th‐century occurrences, (c) 21st‐century occurrences. See data in Appendix S2, Table A2.3, outline of subareas in Figure A2.3. Map is projected in EPSG Projection 4,326—WGS 84

The Tiligul Liman is an 80 km long estuary that is up to 19 m deep (Figure 8). It was disconnected from the Black Sea in the 18–19th century due to the formation of a coastal barrier, but a canal still provides limited water exchange. In the 1960s, the liman contained freshwater and brackish mesohaline zones. However, salinity increased after the construction of a canal, combined with excessive evaporation. The Tiligul Liman drainage consists of steppe rivers that dry during the summer and are unsuited for PC species. Historically, Tiligul Liman contained a few PC species. The specific ecological community which used to live here was dominated by PC (e.g., *M*. *colorata)* and marine cardiids (Grinbart, 1953b). However, *D. polymorpha*, *M. colorata*, and the *Theodoxus* spp. that lived in the liman have disappeared as a result of a human‐driven salinity increase (Moroz et al., 1986; Son, 2007b).

#### 4.1.6 Berezan Liman

The Berezan Liman is 26 km long, with a surface area of c 60 km^2^, a maximum depth of 26 m and is connected to the Black Sea by a canal (Figure 8). The liman has many bays that have very different hydrological settings. The Solonets Tuzly Bay became separated and transformed into a hypersaline lake in the 20th century. In several places, dams have been erected to create isolated areas for aquaculture which is impeding water exchange. Most rivers draining into the Berezan Liman are seasonal steppe rivers that dry out during summer. This seasonality renders them unsuitable for PC species with the exception of the lower Berezan River, where *D*. *polymorpha* occurs (Son, 2007b). Salinities within the Berezan Liman historically ranged between about 3–6 psu but were depressed by an influx of low saline waters during peak discharges from the adjacent Dnieper–Bug estuary through a channel connecting the liman to the Black Sea (Grinbart, 1955).

In the earlier part of the 20th century, Berezan Liman was dominated by *M*. *colorata,* as well as *Theodoxus* spp. (Grinbart, 1953b) and further contained *D*. *polymorpha*. In recent times, *M. colorata* has disappeared in several sites it previously occurred, but some areas within the estuary have not been explored (MOS, PO); other PC species still occur in this liman (Son, 2007b).

#### 4.1.7 Dnieper–Bug Estuary

The Dnieper–Bug Estuary contains the South Bug Estuary, Bug River up to Novaya Odessa City and the Dnieper Liman, Delta, and lower Dnieper River up to the Kakhovka Dam (Figure 8). The Dnieper Estuary is 55 km long and on the Black Sea side is limited by a constriction at the north end of the Kinburn Spit. To the south side, the Yagorlyk Bay may also be included in the Dnieper–Bug complex. The Bug estuary is 47 km long and has a maximum depth of 22 m. The central areas have mostly silty bottoms, and the shore zones are mostly sandy with occasional rocky outcrops. Before the 19th century, the Dnieper–Bug estuary had a salinity gradient similar to the Dniester Liman. Within the outer zone, variable salinities occurred with an average 4 psu. However, increased regulation of the river basins and construction of shipping channels resulted in large‐scale changes in the salinity regimes. A hydropower dam construction in the 1950s restricted freshwater input resulting in a strong salinity increase (with freshwater and oligohaline areas badly affected), but also resulted in episodic massive release of freshwater. Afterward, salinities gradually lowered and the initial gradient more or less returned (Shatova et al., 2009). However, a combination of weak river flow and strong western winds has at times, pushed mesohaline Black Sea waters through the Bugsko–Dneprovsko–Lymansky Canal upstream to Mykolayiv and Kherson ports (Dotsenko & Ivanov, 2010). These incursions of marine waters have dramatically changed salinity regimes and increased variability, especially in the narrow Bug Liman.

The Dnieper–Bug Estuary is historically a major center of PC biodiversity in BSB (Figure 4). A diverse PC fauna containing some local endemic species existed here in the early 20^th^ century (Borcea, 1926a, 1926b; Golikov & Starobogatov, 1966, 1972; Grossu, 1956, 1962; Markovsky, 1954a; Milaschewitsch, 1916; Mordukhay‐Boltovskoy, 1960; Scarlato & Starobogatov, 1972). Some PC species, including *C. variabilis,* were recorded in the Yagorlyk Bay on the south side of the Dnieper–Bug Estuary (Anistratenko, 1996) and *L*. *lincta* in the upper Dnieper Delta near Kherson (Wilke et al., 2007). The Dnieper Liman has been severely affected by the construction of a cascade of dams along the Dnieper River which has led to the severe decline of PC communities. Pontocaspian communities only remained in the eastern part of the liman adjacent to the delta (Moroz & Alexenko, 1983). According to our observations (VVA: 2016–2019), the range of PC communities also decreased in the estuarine part of the southern Bug (upper South Bug Liman and lower South Bug River). Communities declined and some species became very rare or went locally extinct such as *A. fragilis*, *H. plicata*, *Turricaspia chersonica*, and *Clathrocaspia knipowitchii*.

Since the construction of the cascade of reservoirs on the Dnieper River in the 1930–1970s, the water flow rate decreased markedly and the accumulation of silt increased. Algal blooms have become more frequent in the reservoirs and estuaries of the Dnieper and the bottom oxygen content has decreased leading to local anoxic conditions (Romanenko, 1987; Zakonnov et al., 2019). Together with progressive siltation at the bottom of reservoirs, areas of hard substrates, on which *Dreissena* associations and communities of higher aquatic vegetation can occur, were reduced too (e.g., Alexenko & Shevchenko, 2016). This resulted in a gradual, but widespread reduction of habitats suitable for PC gastropod species, such as *Clathrocaspia* spp. that rely on dreissenid bivalves to deposit their eggs (Alexenko & Kucheryava, 2019; Alexenko & Shevchenko, 2016).

#### 4.1.8 Taganrog Bay–Don Delta

**FIGURE 9:**
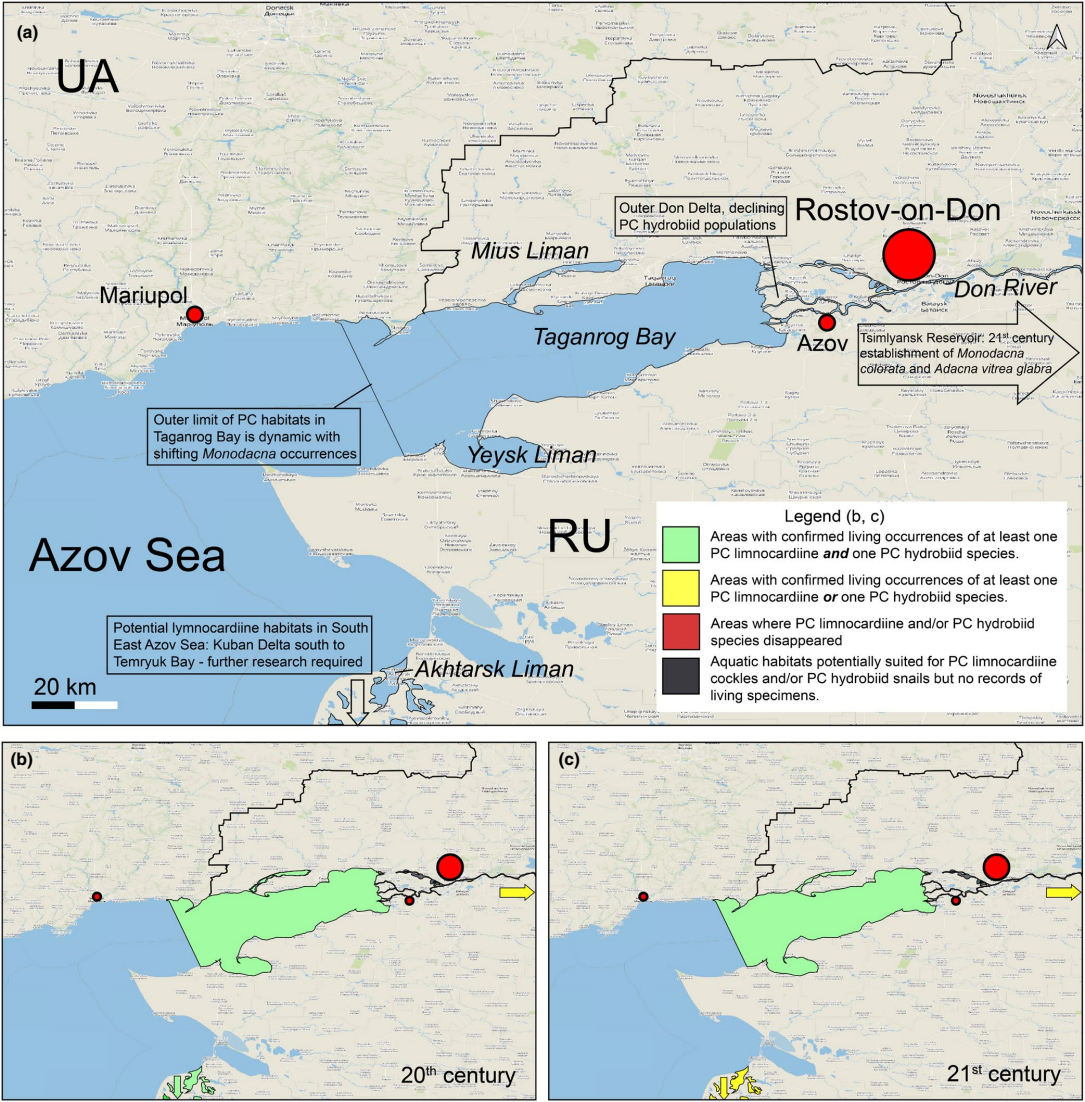
Pontocaspian habitats and trends in the Taganrog Bay–Don Delta region. (a) Regional overview and major trends, (b) 20th‐century occurrences, (c) 21st‐century occurrences. See data in Appendix S2, Table A2.4, outline of subareas in Figure A2.4. Map is projected in EPSG Projection 4,326—WGS 84

The Taganrog Bay, adjacent Mius and Yeysk limans, and the Don River Delta (Figure 9) form the main PC biodiversity hot spot in the northeastern BSB with a rich fauna and different types of PC‐dominated communities (Mordukhay‐Boltovskoy, 1960). Taganrog Bay is a large (5,600 km^2^) and shallow (0–2 m depth in the eastern part and down to 9–10 m in the west) bay (Zhidkova et al., 2018, Ecological Atlas, 2019). It hosts a major salinity gradient from mostly freshwater at its eastern end, to 8–15 psu at the western end. Pontocaspian communities flourish in freshwater to lower mesohaline settings (0–5 psu) in areas with occasional fluctuations of salinities up to 8 psu. The bay floor is mostly silty in the central areas and sandy along the margins, where shell accumulations are also sometimes common. Near large ports (e.g., Taganrog, Mariupol, and Yeysk), black, jelly‐like anthropogenic sediments with high concentrations of petrochemicals and other pollutants occur (Bespalov, 2005). The upper sediment layer in the bay is commonly disturbed by storm waves. The wind is a major factor determining water circulation and therefore salinity distribution in the bay (Matishov & Grigorenko, 2017). Strong western storms can push mesohaline waters to the eastern end of the bay and even occasionally flood the adjacent Don Delta with 4–5 psu waters (Matishov & Grigorenko, 2017). Other drivers affecting the salinity gradients in the bay are the river flow volume and Black Sea water advections (Matishov & Grigorenko, 2017). Two large limans adjoin the bay approximately in its middle. The Mius Liman (33–40 km long and only 1 m deep: Vishnevetskiy & Popruzhniy, 2018) to the north is a drowned estuary with average salinities between 0.9 and 1.8 psu (Kreneva et al., 2013), while the Yeysk Liman to the south is an open estuary with hydrological conditions similar to the adjacent Taganrog Bay. The benthic fauna is different here due to small nature of this water body (Nabozhenko & Kovalenko, 2011). The Don is a regulated river with a mostly sandy bottom. It has some very deep pits (down to 22 m deep) where PC biota occur but, to date, no PC mollusks have been mentioned.

The Inner Taganrog Bay hosts *Dreissena* and *Monodacna* communities. *Adacna fragilis* is also common. In the outer delta areas, a rich PC fauna of 11 species occurred until recently together with freshwater species, for example, unionid mussels, planorbid snails, and *Lithoglyphus naticoides*. The outer delta–bay transitional zone hosts the only known occurrences of the extremely rare *Clathrocaspia logvinenkoi* (Anistratenko, 2007b). Historically, PC species were common in the Taganrog Bay and the outer Don River Delta. In early 2000, communities were changing (Shokhin et al., 2006) but later works showed the persistence of, slightly altered but nevertheless diverse, *M*. *colorata* communities in the inner and central bay area (Nabozhenko, 2008) and the Yeysk Liman (Nabozhenko & Kovalenko, 2011).

Until recently, Taganrog Bay remained relatively unaffected by invasive species. However, the introduction of three exotic polychaete species in 2013–2015 resulted in considerable changes in the bottom communities of the Taganrog Bay and the Don Delta by 2017–2018 (Bick et al., 2018; Syomin et al., 2017). Within a few years after introduction, the alien polychaete *Marenzelleria neglecta* became dominant in the PC habitats in the eastern part of the Taganrog Bay. However, its sharp increase has not been associated with considerable shifts in *Monodacna* abundance or species structure of corresponding communities thus far. *Corbicula* cf. *fluminea,* which was first found in the Don River in 2017 (Zhivoglyadova et al., 2018), is considered one of the most aggressive invasive species tending to lead to negative environmental consequences (Bespalaya et al., 2018; Crespo et al., 2015) and is therefore likely to be a hazardous exotic species for PC mollusks in the freshwater and oligohaline zones. Recently, the brackish water mussel *M*. *leucophaeta* was reported from the inner Taganrog Bay (Zhulidov et al., 2015), which, if capable to survive low winter temperatures, can disrupt PC habitats, as has been reported in the Dniester Liman.

The Taganrog Bay and the Don River are located in a densely populated area with intensive shipping, agricultural, and industrial activity. Dredging and dumping are common in the eastern parts of Taganrog Bay where artificial fairways are subject to permanent siltation. Continuous dredging also occurs in the Don River, especially in the delta. The Lower Don and the Taganrog Bay waters are strongly eutrophicated due to the sewage discharge and terrigenous nutrients from agricultural fertilizers (Matishov, 2005; Moses et al., 2012). Large industrial ports (e.g., Taganrog and Mariupol) are sources of local toxic contamination as well. A considerable threat is the Bagayevskiy waterwork facility which is planned to be put into operation in 2023 (http://bguzel.ru/). According to preliminary estimates, the waterworks will lead to wide‐scale changes in the Lower Don ecosystem (Dubinina & Zhukova, 2016; Krivoshey, 2016).

#### 4.1.9 South East Azov Sea coast

The South East Azov Sea coast includes the coastal zone of Temryuk Bay, northwards to Primorsko–Akhtarsk and the estuaries and channels of the Kuban Delta. The marine part has typical features of the southern Azov Sea, with mesohaline conditions and faunas, sandy beaches, and silty and shelly sediments at depths over 2 m (Simonov & Altman, 1991). The estuaries and channels of the Kuban Delta contain waters from fresh to lower mesohaline conditions and are mostly shallow (average depth within 0.5–1.8 m), with various bottom sediments (e.g., silt, shells, and sand; see Nagalevsky & Nagalevsky, 2013). Little recent information is available on the PC species occurrences from South East Azov Sea coast. *Monodacna colorata* was recorded in environmental impact assessments for oil exploration from the Kurchanskiy, Konovalovskiy, Kulikovskiy, and Polyakov limans (Korpakova et al., 2007) and the Temryuk Bay itself (Korpakova et al., 2008). Also, *D*. *polymorpha* communities, with relatively high biomass, were mentioned across the area as a dominant species (Korpakova et al., 2010). No recent records of PC hydrobiid species are known from the region, even though their general presence in the area was reported by Golikov and Starobogatov (1972).

As the PC species occurrences are poorly known, we have no insights into their trends, but the area is subject to severe anthropogenic modifications. These include invasive species (Syomin et al., 2020), oil/gas exploration and production in Temryuk Bay (Nagalevsky & Lobko, 2017), and the shallowing and siltation in the estuaries of the Kuban Delta area resulting from hydraulic engineering and pollution by the drainage waters from rice fields. Some limans have been transformed in aquaculture ponds losing PC habitats.

#### 4.1.10 Tsimlyansk Reservoir

A recent expansion of *M*. *colorata* and *A*. *vitrea glabra* upstream into the Tsimlyansk Reservoir in the Don River has been documented by Son et al. (2020). The latter species was imported through ballast water by ship traffic from the Caspian Sea through the Volga–Don Canal. *Monodacna colorata* expanded from Taganrog Bay and has now moved through the Volga–Don Canal upstream in the Volga River (AFS and MVV, PO 2017). Species‐rich *Dreissena* communities, with high biomass, containing PC crustaceans, bryozoans, polychaetes, and hydrozoans are common on hard and sandy substrata in the reservoir (Bulysheva et al., 2019; VLS, PO 2018).

### 4.2 Threats

Five direct threats have been shown or postulated to drive the decline of PC communities and species (for references, see below). These are as follows: (a) damming of rivers, (b) modification of marine and freshwater influx in coastal areas, (c) invasive alien species, (d) pollution/eutrophication, and (e) climate change.

#### 4.2.1 Damming of rivers

Damming of rivers (IUCN threat category 7.2 Dams & water management/use) is common in almost all major PC rivers. The construction of dams and large‐scale water irrigation systems resulted in modifications of river flow regimes that affected PC species and communities (Lyashenko et al., 2012; Semenchenko et al., 2015; Son, 2007b). Many PC species are sensitive to oxygen availability and river flow regimes (Mordukhay‐Boltovskoy, 1960). The newly built structures, such as cascades at reservoir dams and cement‐lined canals and riverbanks, have provided new habitats for some *Theodoxus*/*Dreissena* species (Semenchenko et al., 2015, 2016; Son, 2007b). At the same time, soft‐bottom or vagile species that are dependent on intermittent flow regimes (e.g., hydrobiids) declined with the newly erected barriers (Son, 2007a). In river networks, the damming resulted in compartmentalization and disappearance of small river basins and the degradation of floodplains and deltas of larger rivers. Within the estuaries, damming has led to isolation and local salinization, resulting in a reduction in prime PC habitat. Silt accumulation, which causes the loss of hard substrate and vegetation (as a result of restricted river flow by damming) has created adverse conditions for PC communities in the Dnieper River (Romanenko, 1987; Zakonnov et al., 2019). These adverse conditions have resulted in declining habitat (Alexenko & Kucheryava, 2019; Alexenko & Shevchenko, 2016). Such deterioration also applies to other rivers of the NW Black Sea region (South Bug, Dniester), as well as the lower Don River and Taganrog Bay (Anistratenko et al., 2011; Shokhin et al., 2006). Siltation should be considered as an important, perhaps even a key factor triggering habitat reduction threatening PC biota.

#### 4.2.2 The modification of marine and freshwater influx in coastal areas

Modification of marine and freshwater influx in coastal areas (IUCN threat category 7.3 Other ecosystem modifications) affects natural salinity regimes and gradients that sustain(ed) PC species and communities in the coastal zone. It concerns (a) restriction of Black Sea water input through coastal barrier erection and closing of inlets, (b) increasing freshwater influx through diversion canals from adjacent rivers, (c) increased river discharge variability as a result of upstream water withdrawal and episodic release (worsened by increased summer droughts and peak flooding), and (d) increased marine influx through the construction and dredging of shipping lanes and breaching of coastal barriers. Each region contains a specific combination of factors affecting salinity gradients and regimes that sustain PC species and communities, but overall, the variability has strongly increased. In many of the PC areas, (episodic) influx of mesohaline Black Sea waters increased as a result of canal construction and dredging. For example, deep‐water shipping canals, that require regular dredging, resulted in massive seawater intrusion into estuaries and river deltas during storm surges causing rapid salinity fluctuations. The impact may be magnified due to large‐scale water withdrawal upstream from these estuaries and river deltas. In several regions, breaching of sand barriers and spits resulted in strong salinity increases and the breakdown of the pre‐existing stable gradients (Mikhailov & Gorin, 2012). Other estuaries and bays have become isolated hypersaline lakes as a result of their separation from the major limans, either by natural or by man‐made interventions (Vinogradov et al., 2014). These hypersaline lakes (including the entire Tiligul Liman) are hostile to PC species. The breakdown of salinity gradients in Danube coastal lake systems, due to the closing of Black Sea inlets and river diversion, has been a major factor driving the demise of PC species and communities there (Son, 2007b; Velde et al., 2019). Pontocaspian species in the nontidal BSB estuaries live across wide salinity gradients but often occur in the relatively constant salinity regimes of the bottom water layers (Khlebovich, 1974). Populations of PC species have local acclimatization optima and are negatively affected by rapid salinity fluctuations even when occurring within the limits of their autecological tolerance (Orlova, 1987; Orlova et al., 1998; Zhulidov et al., 2018). Increasing salinity variability is especially beneficial to generalist alien and native species (Shiganova, 2011; Zhulidov et al., 2018).

#### 4.2.3 Invasive alien species

Invasive species (IUCN threat category 8.1 Invasive non‐native/alien species/diseases) are an ongoing concern for PC biota (Alexandrov et al., 2007; Bij de Vaate et al., 2002; Son, 2007a). Pontocaspian communities have been replaced by communities dominated by invasive *Mytilopsis leucophaeata, P. antipodarum, Rhithropanopeus harrisii,* and other euryhaline species in the outer part of the Dniester Liman and upper Bug‐Ingul estuarine zone in areas previously inhabited by *Clessiniola*, limnocardiine, and other PC species (Son, 2008; Son et al., 2013; Zhulidov et al., 2018). Community turnover can be very rapid, as shown by Syomin et al. (2017), for the Taganrog Bay. In some of the lower estuaries, increased salinity has resulted in the replacement of PC communities by marine communities, which have colonized these areas from the Black Sea (Zhulidov et al., 2018). These marine communities are heavily affected by three invasive mollusk species, especially in the NW Black Sea: *Mya arenaria*, *Rapana venosa,* and *Anadara* sp. (see for taxonomy discussion of the latter Anistratenko et al., 2014; Anistratenko & Khaliman, 2006; Krapal et al., 2015). In areas with strong freshening, such as the Razim–Sinoe system, freshwater mollusk species, including non‐native bivalves (i.e., *S*. *woodiana*, *C*. *fluminea*) and viviparids, expanded at the cost of PC species (Popa & Murariu, 2009; Velde et al., 2019). Some PC species have become invasive themselves. The Quagga mussel, *D*. *bugensis*, expanded in the second half of the 20th century from its native NW BSB range into all PC habitats, major westerncentral European inland water systems and even freshwater ecosystems in North America (Lyashenko et al., 2012; Son, 2007a, 2007b). The BSB species *M*. *colorata* has recently been introduced into the Volga River and the Caspian Sea, as well as Lake Balkhash–Kazakhstan (Son et al., 2020; Wesselingh et al., 2019). A native Caspian subspecies, *A. vitrea glabra,* recently expanded into the Don River drainage and has a large impact on local benthic species and communities (Son et al., 2020). Increased shipping activity between the Volga and Don River systems has increased the introduction risk of Caspian PC species in the BSB.

#### 4.2.4 Pollution and eutrophication

Pollution and eutrophication (IUCN threat categories 9.3.1 Nutrient loads, 9.3.3 Herbicides & pesticides, 9.6.2 Thermal pollution) are rampant throughout the region, resulting from large‐scale industrial and agricultural activities in the BSB river systems (Lyashenko et al., 2012; Semenchenko et al., 2015). Organic pollution and eutrophication negatively affect PC communities and species that are sensitive to oxygen regimes (Mordukhay‐Boltovskoy, 1960; Popa et al., 2009). Thermal pollution is a local threat to Kuchurgan Estuary and the lower Dnieper River by simultaneously affecting the PC communities and creating preferable conditions for alien species (Protasov et al., 2013; Son, 2007a; Son et al., 2013). Eutrophication has been proposed as a driver for the demise of lymnocardiine species in many lakes in the Danube Delta area (Popa et al., 2009) and also appears to negatively affect communities in Lake Sasyk at the northern end of the Danube Delta, yet pollution levels in the Razim–Sinoe system were found to be low (Catianis et al., 2018).

#### 4.2.5 Climate change

The direct impact of climate change (IUCN threat categories 11.1 Habitat shifting & alteration, 11.2 Droughts, 11.4 Storms & flooding) on PC communities and habitats has been demonstrated in the BSB. In the Taganrog Bay, the influx of mesohaline Black Sea waters increased as a result of a shortage of freshwater inflow due to insufficient river flow regulation linked to climate change (Matishov et al., 2017). Increased summer droughts and peak flooding are making inflowing river discharge more unpredictable. During prolonged summers, rivers may even cease to deliver freshwater to the PC habitats. This is already affecting areas within the Dniester and Dnieper regions and the Tiligul and Berezan limans. Projected climate change with higher temperatures, increased periodic drought, and very high peak discharge in the catchments can be expected to further increase the instability of PC habitats. Additionally, projected rises in sea level will affect coastal lagoons and estuaries (Velde et al., 2019).

## 5 DISCUSSION—TOWARD EFFECTIVE CONSERVATION OF PONTOCASPIAN BIOTA IN THE BLACK SEA BASIN

The combined evidence of this review paper indicates a decline of PC mollusk species and their communities throughout the BSB. However, while the decline seems evident, its ecological consequences are not. It is largely unknown to what extent the species associated with the PC taxa (e.g., their parasites or predators) may be affected by their demise. The decline in abundance and apparent fragmentation (and isolation) of populations is a problem in itself, but may drive genetic depletion, which should also be another reason for concern. Data on genetic diversity of PC species in the BSB are scarce, and little understanding exists on patterns and processes of gene flow between populations, even though it may be an important determinant of PC biodiversity maintenance (Audzijonyte et al., 2006, 2017).

The first step toward effective conservation is improving (a) scientific knowledge on PC biodiversity at community, species, and genetic levels and (b) understanding population and community dynamics as well as species distributions and their ecological tolerances (Cardoso et al., 2011). Recurring and standardized collection and observation efforts are paramount as a basis for establishing trends. These efforts shall be cross‐country collaborative efforts given the transnational character of the PC species and habitats. Furthermore, an improved taxonomical base from integrated morphological‐genetic studies is required, whenever the limited amount of living specimens allow for such approaches. Such studies should extend beyond mollusk species and include other groups of PC invertebrate and vertebrate taxa. For many important PC invertebrate groups (such as copepods, amphipods, and decapods), no up‐to‐date taxonomic overview exists and they contain disputed species (Table 2). Historical distribution data are often imprecise and also hampered by uncertainty in identifications (see Appendix S1). Updated taxonomy will enable targeted research into autecological tolerances and species responses to disturbances. Additionally, the extinction risk of species should be updated through IUCN assessments, as many of the taxa concerned are currently data deficient to perform such analyses (see Wesselingh et al., 2019). New data on PC populations, species, and communities will enable a more inclusive and comprehensive definition of PC habitats and their inclusion in conservation schemes.

**TABLE 2:** Approximate species richness for various invertebrate PC groups in the BSB

PC group	Number of species	Author
Cnidaria	2–4 spp.	Mordukhay‐Boltovskoy (1960)
Crustacea–Amphipoda	40–45 spp.	Mordukhay‐Boltovskoy (1960)
Crustacea–Copepoda	12 spp.	Monchenko (2003)
Crustacea–Cumacea	11 spp.	Mordukhay‐Boltovskoy (1960)
Crustacea–Decapoda	2 spp.	Policar et al. (2018)
Crustacea–Mysidae	9 spp.	Audzijonyte et al. (2008)
Hirudinea	1 sp.	Mordukhay‐Boltovskoy (1960)
Mollusca–Bivalvia	6 spp.	This work
Mollusca–Gastropoda	10 spp.	This work
Polychaeta	3 spp.	Kiseleva (2004)

Secondly, our proposed optimum PC habitats shall be validated using the quantitative data on up‐to‐date PC population sizes and standardized threat analyses shall be performed such as those conducted by Lattuada et al. (2019) for the Caspian Sea and Birstein et al. (2006) and Vassilev (2006) for sturgeon habitats. Threat analyses should focus on four PC regions in the BSB (Danube Delta–Razim Lake system, Dniester Liman, Dnieper–South Bug Estuary, and Taganrog Bay–Don Delta) that contain target species and environmental conditions which can and in cases do support the survival of PC communities (Table 1, Figure 2). Quantitative knowledge on population sizes of PC species is lacking for both mollusks and other groups. For example, crustaceans contain large numbers of PC species (Table 2) and their inclusion would greatly improve the definition of optimum PC habitats. Our proposed optimum PC habitats are therefore indicative for the moment.

The final step should be assessing some of the indirect anthropogenic drivers of PC biodiversity change that are causing the identified direct drivers of decline, such as institutional arrangements and legal landscape, following the IPBES Conceptual Framework (Díaz et al., 2015). Institutional alignment and responsibilities to address PC biodiversity conservation and governance have been studied by Gogaladze, Raes, et al. (2020), Gogaladze, Wesselingh, et al. (2020) who showed that this biota is not a priority for conservation planning in Ukraine and Romania. Future studies are required to understand legal arrangements of countries sharing the PC biodiversity and their outcomes for conservation. Currently, some parts of optimum PC habitats are covered by national and/or large transnational protected areas such as the Danube Delta Biosphere Reserve shared by Ukraine and Romania. Other parts are covered by Emerald sites (https://emerald.eea.europa.eu/), Natura 2000 sites (https://Natura2000.eea.europa.eu/) and/or by Ramsar sites (https://www.protectedplanet.net/166893). The coverage of optimum PC habitats by protected areas may provide (incidental) protection to PC communities and species, but has not resulted in targeted conservation to date. Assignment of optimum PC habitats to IUCN category IV: habitats/species management area (Dudley, 2008) can be a useful approach. The IUCN protected area management categories provide a global framework for sorting the variety of protected area management aims. Category IV aims to “maintain, conserve and restore species and habitats” (https://www.iucn.org/theme/protected‐areas/about/protected‐areas‐categories/category‐iv‐habitatspecies‐management‐area). Such categorization can take place in different phases of establishing a protected area, such as the initial phase: before the protected area is established and category has to be decided, or in later phase: after the protected area has already been established and category decided, but management aim is to address emerging conservation priorities (Dudley, 2008). Managing and mitigating the wholesale decline of the unique PC biota in the BSB will require long‐standing commitment from various stakeholders across countries bordering the Black Sea.

## 6 CONCLUSIONS

Pontocaspian mollusk species and communities in the BSB have suffered a severe decline over the past century. Five major drivers for the decline are identified. However, basic distribution data and integrated approaches to mitigate the decline are lacking. Some PC communities have already vanished and many species have gone extinct or are under increased risk of extinction. The identification of optimum PC habitats will enable targeted conservation actions. Sustained, transnational collaboration is required to improve conservation of PC species, communities, and their habitats in the BSB. Only then can the effective conservation of the unique and threatened PC biota be achieved in the region.

## CONFLICT OF INTEREST

The authors have no conflict of interest.

## AUTHOR CONTRIBUTION

**Aleksandre Gogaladze:** Conceptualization (lead); Formal analysis (lead); Methodology (lead); Software (equal); Validation (equal); Visualization (equal); Writing‐original draft (equal); Writing‐review & editing (equal). **Mikhail O. Son:** Conceptualization (equal); Formal analysis (equal); Methodology (equal); Software (equal); Validation (equal); Visualization (equal); Writing‐original draft (equal); Writing‐review & editing (equal). **Matteo Lattuada:** Methodology (equal); Software (equal); Validation (equal); Visualization (equal); Writing‐review & editing (equal). **Vitaliy V. Anistratenko:** Formal analysis (equal); Methodology (supporting); Validation (equal); Visualization (supporting); Writing‐original draft (supporting); Writing‐review & editing (supporting). **Vitaly L. Syomin:** Formal analysis (equal); Validation (equal); Visualization (supporting); Writing‐original draft (supporting). **Ana Bianca Pavel:** Formal analysis (equal); Validation (equal); Visualization (supporting); Writing‐original draft (supporting). **Oana P. Popa:** Formal analysis (equal); Validation (equal); Visualization (supporting); Writing‐original draft (supporting). **Luis O. Popa:** Formal analysis (equal); Validation (equal); Visualization (supporting); Writing‐original draft (supporting). **Jan‐Johan ter Poorten:** Formal analysis (supporting); Validation (equal); Visualization (supporting); Writing‐original draft (supporting). **Jacobus C. Biesmeijer:** Conceptualization (equal); Validation (equal); Writing‐review & editing (supporting). **Niels Raes:** Conceptualization (equal); Validation (equal); Writing‐review & editing (supporting). **Thomas Wilke:** Conceptualization (equal); Formal analysis (equal); Methodology (supporting); Validation (equal); Visualization (supporting); Writing‐review & editing (supporting). **Arthur F. Sands:** Formal analysis (equal); Validation (equal); Visualization (supporting); Writing‐review & editing (supporting). **Teodora Trichkova:** Formal analysis (equal); Validation (equal); Visualization (supporting); Writing‐original draft (equal); Writing‐review & editing (supporting). **Zdravko K. Hubenov:** Formal analysis (equal); Validation (equal); Visualization (supporting); Writing‐original draft (equal). **Maxim V. Vinarski:** Formal analysis (equal); Validation (equal); Visualization (supporting); Writing‐original draft (equal). **Olga Yu Anistratenko:** Formal analysis (equal); Methodology (supporting); Validation (equal); Visualization (supporting); Writing‐original draft (equal); Writing‐review & editing (supporting). **Tatiana L. Alexenko:** Validation (equal). **Frank P. Wesselingh:** Conceptualization (equal); Formal analysis (equal); Methodology (equal); Software (equal); Validation (equal); Visualization (equal); Writing‐original draft (equal); Writing‐review & editing (equal).

## Supporting information

Appendix S1

Appendix S2

Appendix S3

## Data Availability

All data that support the findings of this study are provided in appendices. Pontocaspian habitat polygon shapefiles and the attributes describing historical (20th century) and modern (21st century) distributions of PC target taxa are available on Dryad, https://datadryad.org/stash/share/cMhMU‐zTUUULuZM1XjtQKZNwN5M‐L6cwKiKP4kaf6go.
