# Supplementary material for: Decline of unique Pontocaspian biodiversity in the Black Sea Basin: A review
Source: Ecol Evol. 2021 Sep 7;11(19):12923–47. doi: 10.1002/ece3.8022 (PMC8495785; doi:10.1002/ece3.8022)
Supplement: Supplementary file 1 — Appendix S1 [file ECE3-11-12923-s003.docx]

**Appendix S1 – Update on the taxonomic status of Black Sea Basin Pontocaspian mollusc species**

Wesselingh et al. (2019) discussed the Pontocaspian (PC) mollusc species list, including species whose status at the time were agreed upon (“accepted”) and those whose status was considered to be uncertain, based on a review of existing data. Currently several taxonomic studies have been completed or are under way that will provide further clarification. Here a brief summary of the taxonomy of the Black Sea Basin (BSB) PC mollusc species is given.

Neritidae – The recent revision of *Theodoxus* species (Sands et al., 2019) clarified the species delimitation of this genus and showed the presence of four *Theodoxus* species in the BSB (*T. danubialis*, *T. fluviatilis*, *T. velox* and *T. major*. The naming of latter, often listed as *T. pallasi* was discussed by Wesselingh et al. (2019) and more recently by Sands et al. (2020). They showed that discrimination based on shell morphology is not always sufficient and that some historical records should be re-evaluated. Especially, the confirmation of *T. major* and *T. velox* occurrences in the BSB can be expected to change with further study.

Hydrobiidae – The taxonomic status of PC hydrobiid snails is subject of a number of ongoing studies that will lead to further clarification for species boundaries (Anistratenko et al., 2021). A molecular study on the identity of BSB *Clathrocaspia* species is currently under way to assess the status and potential synonymy of four species listed by Wesselingh et al. (2019) (TW & VVA, pers. comm.). The status of two of the smooth PC hydrobiid species listed from the BSB by Wesselingh et al. (2019) (?*Laevicaspia* *ismailensis* (accepted) and *Turricaspia chersonica* (uncertain)) requires further study involving molecular analyses and study of type material to assess possible conspecificity and establish the generic assignemnt (VVA, pers. comm.). The status of *Laevicaspia lincta* and *Clessiniola variabilis* is undisputed.

Cardiidae – After Wesselingh et al. (2019) published the PC species list, further material of BSB *Adacna fragilis* has been inspected in order to assess its status (listed as uncertain in the publication). The range of morphological variation of the BSB material (especially the almost equilateral shell, the pallial sinus not extending the vertical midline, the more pronounced and well demarcated ribs and the smaller adult size), as well as the salinity preferences differ from the resembling Caspian *Adacna* *laeviuscula* and merits a separation of the two species. There is full agreement to change the status of *Adacna fragilis* to accepted species among the authors. The species *Adacna glabra* reported by Son et al. (2020) from the Don River was considered as an uncertain status subspecies of *Adacna vitrea* by Wesselingh et al. (2019), who argued for molecular confirmation. However, a review of the distribution range, ecological tolerance and shell characters shows that it is likely that *A. glabra* is closely related to, but at the same time distinct from *A. vitrea*. *Adacna glabra* differs by having somewhat stronger developed ribs with a rather pointed rib crest and the often whitish colour of the shell. We adopt for the moment the distinction proposed by Kijashko (Kijashko in Bogutskaya et al., 2013) and consider *A. vitrea vitrea* and *A. vitrea glabra* as subspecies whose status will need molecular corroboration.

Dreissenidae – The taxonomy and status of the two BSB dreissenid species (*Dreissena* *polymorpha* and *D. bugensis*) is undisputed.

**References**

Anistratenko, V. V., Neubauer, T. A., Anistratenko, O. Y., Kijashko, P. V. & Wesselingh, F. P. 2021. A revision of the Pontocaspian gastropods of the subfamily Caspiinae (Caenogastropoda: Hydrobiidae). *Zootaxa,* 4933**,** 151-197.

Bogutskaya, N. G., Kijashko, P. V., Naseka, A. M. & Orlova, M. I. 2013. Identification keys for fish and invertebrates. Volume 1: Fish and molluscs. KMK Scientific Publishers, Moscow. [in Russian].

Sands, A. F., Glöer, P., Gürlek, M. E., Albrecht, C. & Neubauer, T. A. 2020. A revision of the extant species of *Theodoxus* (Gastropoda, Neritidae) in Asia, with the description of three new species. *Zoosystematics and Evolution,* 96**,** 25-66.

Sands, A. F., Sereda, S. V., Stelbrink, B., Neubauer, T. A., Lazarev, S., Wilke, T. & Albrecht, C. 2019. Contributions of biogeographical functions to species accumulation may change over time in refugial regions. *Journal of Biogeography,* 46**,** 1274-1286.

Son, M. O., Prokin, A. A., Dubov, P. G., Konopacka, A., Grabowski, M., MacNeil, C. & Panov, V. E. 2020. Caspian invaders vs. Ponto-Caspian locals – range expansion of invasive macroinvertebrates from the Volga Basin results in high biological pollution of the Lower Don River. *Management of Biological Invasions,* 11**,** 178-200.

Wesselingh, F. P., Neubauer, T. A., Anistratenko, V. V., Vinarski, M. V., Yanina, T., ter Poorten, J. J., Kijashko, P., Albrecht, C., Anistratenko, O. Y., D’Hont, A., Frolov, P., Gandara, A. M., Gittenberger, A., Gogaladze, A., Karpinsky, M., Lattuada, M., Popa, L., Sands, A. F., Velde, S. v. d., Vandendorpe, J. & Wilke, T. 2019. Mollusc species from the Pontocaspian region – an expert opinion list. *ZooKeys,* 827**,** 31-124.
