# Supplementary material for: Decline of unique Pontocaspian biodiversity in the Black Sea Basin: A review
Source: Ecol Evol. 2021 Sep 7;11(19):12923–47. doi: 10.1002/ece3.8022 (PMC8495785; doi:10.1002/ece3.8022)
Supplement: Supplementary file 2 — Appendix S2 [file ECE3-11-12923-s001.docx]

**Appendix S2. Pontocaspian species occurrences in the Black Sea Basin in 20^th^ and 21^st^ centuries.**

Table A2.1. Sub-areas within the Danube-Razim and Bulgarian coastal wetlands. ID numbers of the sub-areas are used in Figures A2.1-4 for display (see below). + indicates the presence and – the absence of the relevant Pontocaspian (PC) mollusc target taxa in 20^th^ and 21^st^ centuries. C stands for century. PO stands for personal observation. Pontocaspian habitat map of the Danube Delta region (Fig. 6 in the manuscript) and the Danube Delta shapefile (Appendix 3) are based on the data in this table.

| Country | ID | Sub-area | PC Cardiidae 20^th^ C | PC Hydrobiidae 20^th^ C | PC Cardiidae 21^st^ C | PC Hydrobiidae 21^st^ C | Remarks |
| --- | --- | --- | --- | --- | --- | --- | --- |
| UA | 1 | Lake Kagul | + Markovsky (1955) | + Markovsky (1955) | + Dzhurtubaev et al. (2017), Munasypova-Motyash (2006) | - Dzhurtubaev et al. (2017) |  |
| UA | 2 | Northern floodplain lakes west of Izmail | - Markovsky (1955) | - Markovsky (1955) | - MOS (PO) | - MOS (PO) | PC taxa (*Dreissena*, *Theodoxus*) present |
| UA | 3 | Lake Yalpug | + Markovsky (1955), VVA (PO) | + Markovsky (1955), VVA (PO) | + Munasypova-Motyash (2006), MOS and VVA (PO) | + MOS (PO) |  |
| UA | 4 | Lake Kugurlui | + Markovsky (1955) | + Markovsky (1955) | + Dzhurtubaev et al. (2018) | - Dzhurtubaev et al. (2018) |  |
| UA | 5 | Lake Katlabukh | + Markovsky (1955) | + Markovsky (1955) | + MOS (PO) | - MOS (PO) |  |
| UA | 6 | Lake Kitai | + Markovsky (1955) | - Markovsky (1955) | - MOS (PO) | - MOS (PO) |  |
| UA | 7 | Lake Sasyk | + Markovsky (1955) | + Markovsky (1955) | + Khalaim and Son (2016), Munasypova-Motyash (2006), MOS and VVA (PO) | - MOS (PO) | Limnocardiid invasion end 20th |
| UA | 8 | Northern floodplain lakes east of Izmail | - Markovsky (1955) | - Markovsky (1955) | - MOS (PO) | - MOS (PO) | PC taxa (*Dreissena*, *Theodoxus*) present |
| ROU, BG | 9 | Upstream Danube River, Brailla-Gura Vai section | - Ignat et al. (1997), Vǎdineanu et al. (2000) | - Ignat et al. (1997), Vǎdineanu et al. (2000), + Russev (1966) - likely present (dead shells) | - ICPDR (2008), ICPDR (2015), Stoica et al. (2013), Stoica et al. (2012), Stoica et al. (2014), ABP (PO) | + Boeters et al. (2015), ICPDR (2008), ICPDR (2015), Stoica et al. (2013), Stoica et al. (2012), Stoica et al. (2014), - ABP (PO) | PC taxa Romania: (*Dreissena*, *Theodoxus*) present (Drensky, 1947, Russev, 1966, Angelov, 2000, Wohlberedt, 1911), TT (2012-2019, PO), ABP (PO) |
| ROU | 10 | Danube River, Braila-Tulcea section | + Popa et al. (2009) | - Markovsky (1955), Ignat et al. (1997), Vǎdineanu et al. (2000) | - Popa et al. (2009), ICPDR (2008), ICPDR (2015), Stoica et al. (2013), Stoica et al. (2012), Stoica et al. (2014), ABP (PO) | - ICPDR (2008), ICPDR (2015), Stoica et al. (2013), Stoica et al. (2012), Stoica et al. (2014), ABP (PO) | PC taxa (*Dreissena*, *Theodoxus*) present ABP (PO) |
| ROU, UA | 11 | Chilia branch and outer delta lakes, upstream from Vilkovo | + Markovsky (1955) | - Markovsky (1955), - Ignat et al. (1997), Vǎdineanu et al. (2000) | - ICPDR (2008), ICPDR (2015), Stoica et al. (2013), Stoica et al. (2012), Stoica et al. (2014), ABP (PO) | - ICPDR (2008), ICPDR (2015), Stoica et al. (2013), Stoica et al. (2012), Stoica et al. (2014), ABP (PO) | PC taxa (*Dreissena*, *Theodoxus*) present ABP (PO) |
| ROU, UA | 12 | Chilia branch and outer delta lakes downstream from Vilkovo | + Markovsky (1955) | + Markovsky (1955) | + MOS (PO) | - MOS (PO) | PC taxa (*Dreissena*, *Theodoxus*) present ABP (PO) |
| ROU | 13 | Sulina branch of Danube River | + ABP (PO) - likely present (dead shells) | - Ignat et al. (1997), Vǎdineanu et al. (2000) | - ICPDR (2008), ICPDR (2015), Stoica et al. (2013), Stoica et al. (2012), Stoica et al. (2014), ABP (PO) | - ICPDR (2008), ICPDR (2015), Stoica et al. (2013), Stoica et al. (2012), Stoica et al. (2014), ABP (PO) | PC taxa (*Dreissena*, *Theodoxus*) present ABP (PO) |
| ROU | 14 | Sf. Gheorghe branch of Danube River | + Popa et al. (2009) | - Ignat et al. (1997), Vǎdineanu et al. (2000) | - Popa et al. (2009); ICPDR (2008), ICPDR (2015), Stoica et al. (2013), Stoica et al. (2012), Stoica et al. (2014), ABP (PO) | - ICPDR (2008), ICPDR (2015), Stoica et al. (2013), Stoica et al. (2012), Stoica et al. (2014), ABP (PO) | PC taxa (*Dreissena*, *Theodoxus*) present ABP (PO) |
| ROU | 15 | Lake Brates | + Popa et al. (2009) | Data deficient | - Popa et al. (2009) | Data deficient |  |
| ROU | 16 | Lake Jijila | + Popa et al. (2009) | Data deficient | - Popa et al. (2009) | Data deficient |  |
| ROU | 17 | Lake Crapina | + Popa et al. (2009) | Data deficient | - Popa et al. (2009) | Data deficient |  |
| ROU | 18 | Floodplain lakes between Chilia and Sulina branches | - Ignat et al. (1997), Vǎdineanu et al. (2000) | - Ignat et al. (1997), Vǎdineanu et al. (2000) | - ICPDR (2008), ICPDR (2015), Stoica et al. (2013), Stoica et al. (2012), Stoica et al. (2014), ABP (PO) | - Stoica et al. (2014), Stoica et al. (2012), Stoica et al. (2013), ABP (2015, PO) | PC taxa (*Dreissena*, *Theodoxus*) present |
| ROU | 19 | Floodplain lakes between Sulina and Sf. Gheorghe branches | - Ignat et al. (1997), Vǎdineanu et al. (2000) | - Ignat et al. (1997), Vǎdineanu et al. (2000) | - ICPDR (2008), ICPDR (2015), Stoica et al. (2013), Stoica et al. (2012), Stoica et al. (2014), ABP (PO) | - ICPDR (2008), ICPDR (2015), Stoica et al. (2013), Stoica et al. (2012), Stoica et al. (2014), ABP (PO) | PC taxa (*Dreissena*, *Theodoxus*) present ABP (PO) |
| ROU | 20 | Lake Puiu | + Popa et al. (2009) | - ABP (PO) | - Popa et al. (2009), ABP (PO) | - ABP (PO) |  |
| ROU | 21 | Floodplain lakes south to Sf. Gheorghe branch | - Ignat et al. (1997), Vǎdineanu et al. (2000) | - Ignat et al. (1997), Vǎdineanu et al. (2000) | - ICPDR (2008), ICPDR (2015), Stoica et al. (2013), Stoica et al. (2012), Stoica et al. (2014), ABP (PO) | - ICPDR (2008), ICPDR (2015), Stoica et al. (2013), Stoica et al. (2012), Stoica et al. (2014), ABP (PO) | PC taxa (*Dreissena*, *Theodoxus*) present ABP (PO) |
| ROU | 22 | Lake Murighiol | + Popa et al. (2009) | - ABP (PO) | - Popa et al. (2009) | - ABP (PO) |  |
| ROU | 23 | Lake Dranov | + Popa et al. (2009) | - ABP (PO) | - Popa et al. (2009) | - ABP (PO) |  |
| ROU | 24 | Sakhalin area | - Gomoiu and Skolka (1996), Gomoiu and Skolka (1998) | - Gomoiu and Skolka (1996), Gomoiu and Skolka (1998) | - Pavel et al. (2019) | - Pavel et al. (2019) |  |
| ROU | 25 | Musura Bay | - Gomoiu and Skolka (1996), Gomoiu and Skolka (1998) | - Gomoiu and Skolka (1996), Gomoiu and Skolka (1998) | - Pavel et al. (2019) | - Pavel et al. (2019) | PC taxa (*Dreissena*, *Theodoxus*) present Pavel et al., 2019 |
| ROU | 26 | Lake Razim-Golovita | + Popa et al. (2009), Teodorescu-Leonte et al. (1956), Teodorescu-Leonte (1966), Teodorescu-Leonte and Leonte (1969), Teodorescu-Leonte (1977) | + Velde et al. (2019), - Grossu (1986) | + Popa et al. (2009), Paraschiv et al. (2010b), Paraschiv et al. (2010a), Velde et al. (2019), ABP (PO) | + Wilke et al. 2007,  - ABP (PO) | PC taxa (*Dreissena*, *Theodoxus*) present ABP, FPW (PO); PC hydrobiids have only been reported once in 21th century (2003; Wilke et al., 2007) and not found in later years |
| ROU | 27 | Lake Sinoe | + Popa et al. (2009); Teodorescu-Leonte et al. (1956); Teodorescu-Leonte (1966); Teodorescu-Leonte and Leonte (1969); Teodorescu-Leonte (1977) | - Velde et al. (2019) | + Popa et al. (2009), Paraschiv et al. (2010b), Paraschiv et al. (2010a), ABP (PO), Tatiana Begun (2004, PO) | - ABP (PO) |  |
| ROU | 28 | Lake Babadag | + Popa et al. (2009) | Data deficient | - Popa et al. (2009), ABP (PO) | - ABP (PO) |  |
| ROU | 29 | Lake Leahova | + Popa et al. (2009) | Data deficient | - Popa et al. (2009), ABP (PO) | - ABP (PO) |  |
| ROU | 30 | Coastal lakes near Sinoe | Data deficient | Data deficient | Data deficient | Data deficient |  |
| BG | NA | Lake Durankulak | - Kovachev et al. (1999), Angelov (2000) | - Kovachev et al. (1999), Angelov (2000) | - Hubenov (2015), Vidinova et al. (2016) | - Hubenov (2015), Vidinova et al. (2016) | PC taxa *(Dreissena,* *Theodoxus)* present (Kovachev et al., 1999, Angelov, 2000); TT (2010, 2014, PO) |
| BG | NA | Lake Shabla-Ezerets | - Kovachev et al. (1999), Angelov (2000) | - Kovachev et al. (1999), Angelov (2000) | - Hubenov (2015), Vidinova et al. (2016) | - Hubenov (2015), Vidinova et al. (2016) | PC taxa *(Dreissena,* *Theodoxus)* present (Kovachev et al., 1999, Angelov, 2000, Valkanov, 1941); TT (2010, 2014, PO) |
| BG | NA | Lake Beloslav | - Drensky (1947) | + Drensky (1947) | - Hubenov (2015) | - Hubenov (2015) | PC taxa *(Dreissena,* *Theodoxus)* present (Valkanov, 1957, Angelov, 2000, Drensky, 1947) |
| BG | NA | Lake Varna | + Kaneva-Abadjieva (1957) | - Kaneva-Abadjieva (1957) | - Hubenov (2015), Vidinova et al. (2016) | - Hubenov (2015), Vidinova et al. (2016) | PC taxa (*Dreissena*, *Theodoxus*) present (Wohlberedt, 1911, Drensky, 1947, Angelov, 2000) |
| BG | NA | Kamchiya River Mouth and backwaters | - Angelov (2000), Valkanov (1957) | - Angelov (2000), Valkanov (1957) | - Hubenov (2015) | - Hubenov (2015) | PC taxa (*Dreissena*, *Theodoxus)* present (Valkanov, 1957, Angelov, 2000), TT (2010, 2011, 2014, PO) |
| BG | NA | Lake Burgas (Vaya) | - Drensky (1947), Valkanov (1957), Angelov (2000) | - Drensky (1947), Valkanov (1957), Angelov (2000) | - Vidinova et al. (2016), Pandourski (2001) | - Vidinova et al. (2016), Pandourski (2001) | PC taxa (*Theodoxus*) present (Valkanov, 1957, Angelov, 2000) |
| BG | NA | Lake Mandra | - Drensky (1947), Mihailova-Neikova (1961) | + Drensky (1947, L. lincta live, June 1944), Mihailova-Neikova (1961) | - Hubenov (2015), Vidinova et al. (2016) | - Hubenov (2015), Vidinova et al. (2016) | PC taxa (*Dreissena*, *Theodoxus*) present (Valkanov, 1957, Mihailova-Neikova, 1961, Angelov, 2000, Drensky, 1947, Vidinova et al., 2016); TT (2014, PO) |
| BG | NA | Ropotamo River Mouth | - Angelov (2000), Valkanov (1957) | - Angelov (2000), Valkanov (1957) | - Hubenov (2015) | - Hubenov (2015) | PC taxa (*Theodoxus*) present (Valkanov, 1957) |
| BG | NA | Dyavolsko Blato Marsh | - Angelov (2000), Valkanov (1957) | - Angelov (2000), Valkanov (1957) | - Hubenov (2015) | - Hubenov (2015) | PC taxa (*Theodoxus*) present (Valkanov, 1957) |
| BG | NA | Karaagach River Mouth and Marsh | - Angelov (2000), Valkanov (1957) | - Angelov (2000), Valkanov (1957) | - Hubenov (2015) | - Hubenov (2015) | PC taxa (*Theodoxus*) present (Valkanov, 1957, Angelov, 2000) |
| BG | NA | Veleka River Mouth | - Angelov (2000), Valkanov (1957) | - Angelov (2000), Valkanov (1957) | - Hubenov (2015) | - Hubenov (2015) | PC taxa (*Dreissena, Theodoxus*) present (Valkanov, 1957, Angelov, 2000); TT (2009, 2014, PO) |
| BG | NA | Silistar River Mouth | - Angelov (2000), Valkanov (1957) | - Angelov (2000), Valkanov (1957) | - Hubenov (2015) | - Hubenov (2015) | PC taxa (*Theodoxus*) present (Valkanov, 1957) |
| BG | NA | Rezovska Reka River Mouth | - Angelov (2000), Valkanov (1957) | - Angelov (2000), Valkanov (1957) | - Hubenov (2015) | - Hubenov (2015) | PC taxa (*Theodoxus*) present (Valkanov, 1957, Angelov, 2000) |

Table A2.2. Sub-areas within the Dniester Liman. ID numbers of the sub-areas are used in Figures A2.1-4 for display (see below). + indicates the presence and – the absence of the relevant PC mollusc target taxa in 20th and 21st centuries. C stands for century. PO stands for personal observation. Pontocaspian habitat map of the Dniester Liman (Fig. 7 in the manuscript) and the Dniester Liman shapefile (Appendix 3) are based on the data in this table.

| Country | ID | Sub-area | PC Cardiidae 20^th^ C | PC Hydrobiidae 20^th^ C | PC Cardiidae 21^st^ C | PC Hydrobiidae 21^st^ C | Remarks |
| --- | --- | --- | --- | --- | --- | --- | --- |
| UA | 1 | Outer Dniester Liman | + Grinbart (1953a), Markovsky (1953), Son (2007) | + Grinbart (1953a), Markovsky (1953), Son (2007) | + MOS, OYA, VVA (PO) | - MOS, OYA, VVA (PO) | PC taxa (*Theodoxus*) present |
| UA | 2 | Middle-Inner Dniester Liman | + Grinbart (1953a), Markovsky (1953), Son (2007) | + Grinbart (1953a), Markovsky (1953), Son (2007) | + Munasypova-Motyash (2006); MOS, OYA, VVA (PO) | + MOS, OYA, VVA (PO) | PC taxa (*Dreissena*, *Theodoxus*) present |
| UA | 3 | Lower Dniester Floodplain | + Grinbart (1953a), Markovsky (1953), Son (2007) | + Grinbart (1953a), Son (2007) | - MOS, OYA, VVA (PO) | - MOS, OYA, VVA (PO) | PC taxa (*Dreissena*, *Theodoxus*) present |
| UA, MD | 4 | Kuchurgan Liman | + Grinbart (1953a), Markovsky (1953), Son (2007) | + Grinbart (1953a), Markovsky (1953), Son (2007) | + Filipenko (2011), MOS, VVA (PO) | - Filipenko (2011), MOS, VVA (PO) |  |
| MD | 5 | Dniester River from Cioburciu to Dubasari | + Munjiu (2012), Cartea Roșie a Republicii Moldova (2015) | - Balashov et al. (2013) | - Cartea Roșie a Republicii Moldova (2015) | - Balashov et al. (2013) | PC taxa (*Dreissena*, *Theodoxus*) present |

Table A2.3. Sub-areas within the Dnieper-Bug Estuary. ID numbers of the sub-areas are used in Figures A2.1-4 for display (see below). + indicates the presence and – the absence of the relevant PC mollusc target taxa in 20th and 21st centuries. C stands for century. PO stands for personal observation. Pontocaspian habitat map of the Dnieper-Bug Estuary (Fig. 8 in the manuscript) and the Dnieper-Bug Estuary shapefile (Appendix 3) are based on the data in this table.

| Country | ID | Sub-area | PC Cardiidae 20^th^ C | PC Hydrobiidae 20^th^ C | PC Cardiidae 21^st^ C | PC Hydrobiidae 21^st^ C | Remarks |
| --- | --- | --- | --- | --- | --- | --- | --- |
| UA | 1 | Tiligul Liman | + Grinbart (1953b) | - Mordukhay-Boltovskoy, 1960 | - Son (2007) | - MOS (PO) | PC taxa (*Dreissena*, *Theodoxus*) present |
| UA | 2 | Berezan Liman | + Grinbart (1953b) | - Grinbart (1953b) | - Son (2007) | - MOS (PO) | PC taxa (*Dreissena*, *Theodoxus*) present |
| UA | 3 | Bug River upstream from Mykolaiv | + Markovsky (1954) | + Markovsky (1954) | - MOS (PO) | - MOS (PO) | PC taxa (*Dreissena*, *Theodoxus*) present |
| UA | 4 | Dnieper - South Bug Estuary | + Markovsky (1954), Zhadin (1931) | + Markovsky (1954) | + Alexenko (2004), Munasypova-Motyash (2006), MOS, OYA, VVA (PO) | + Alexenko (2004), MOS, OYA, VVA (PO) |  |
| UA | 5 | Lower Dnieper -downstream from Inhulets | + Markovsky (1954) | + Markovsky (1954) | + Alexenko (2004), OYA, VVA (PO) | + Alexenko (2004), Wilke et al. (2007), MOS, OYA, VVA (PO) |  |
| UA | 6 | Lower Dnieper – from Inhulets to Kakhovka Reservoir | + Markovsky (1954), TA (PO) | + Markovsky (1954), TA, VVA (PO) | + TA, VVA (PO) | + TA, VVA (PO) | Common 20st century occurrence of hydrobiids, only sporadic 21st century occurrence of hydrobiids (TA, PO) |
| UA | 7 | Dnieper upstream from Kakhovka Reservoir | + Munasypova-Motyash (2006), VVA (PO) | Data deficient | + Munasypova-Motyash (2006), Semenchenko et al. (2016) | Data deficient | PC taxa (*Dreissena*, *Theodoxus*) present 21st C |
| UA | 8 | Yagorlyk Bay | Data deficient | + Anistratenko (1996) | Data deficient | Data deficient | PC taxa (*Dreissena*, *Theodoxus*) present 21st C |

Table A2.4. Sub-areas within the Taganrog Bay-Don Delta. ID numbers of the sub-areas are used in Figures A2.1-4 for display (see below). + indicates the presence and – the absence of the relevant PC mollusc target taxa in 20th and 21st centuries. C stands for century. PO stands for personal observation. Pontocaspian habitat map of the Taganrog Bay-Don Delta (Fig. 9 in the manuscript) and the Taganrog Bay-Don Delta shapefile (Appendix 3) are based on the data in this table and table A2.5 below.

| Country | ID | Sub-area | PC Cardiidae 20^th^ C | PC Hydrobiidae 20^th^ C | PC Cardiidae 21^st^ C | PC Hydrobiidae 21^st^ C | Remarks |
| --- | --- | --- | --- | --- | --- | --- | --- |
| UA, RU | 1 | Taganrog Bay/outer Don Delta | + Mordukhay-Boltovskoy (1960), Scarlato and Starobogatov (1972), Stark (1960), Vorobyev (1949), Nekrasova (1972) | + Anistratenko (2007), Mordukhay-Boltovskoy (1960), Golikov and Starobogatov (1972) | + Nabozhenko (2005), Shokhin et al. (2006), Nabozhenko (2008) | + Kovalenko (2009) | Hydrobiidae species highly abundant in the Don River Delta, Kalancha channel, deep pit in 2 km upstream from mouth (Kovalenko, 2009) |
| RU | 2 | Mius Liman | + Mordukhay-Boltovskoy (1960) | Data deficient, but given abundant records in 2000 (TW, PO) likely present | + VLS, (2003, 2006, PO) | + Wilke et al. (2007), Frank Riedel (2016, PO) | Likely presence of PC taxa in 20^th^ C given the common occurrence in observations in 2000 |
| RU | 3 | Lower Don up to Manych confluence | + Mordukhay-Boltovskoy (1960) | Data deficient | + Nabozhenko (2008) | Data deficient | PC taxa (*Dreissena*, *Theodoxus*) present 20-21st C (Zhivoglyadova and Frolenko, 2017); VLS, 2017-2019 (PO) |
| RU | 4 | Tsimlyansk Reservoir | + Scarlato and Starobogatov (1972) | - Son et al. (2020) | + Son et al. (2020) | - Son et al. (2020) | PC taxa (*Dreissena, Theodoxus*) present 21st Century (Bulysheva et al., 2019); VLS, 2018-2019 (PO) |

Table A2.5. Sub-areas within the SE Azov Sea coast. ID numbers of the sub-areas are used in Figures A2.1-4 for display (see below). + indicates the presence of the relevant PC mollusc target taxa in 20^th^ and 21^st^ centuries. C stands for century. Pontocaspian habitat map of the Taganrog Bay-Don Delta (Fig. 9 in the manuscript) and the Taganrog Bay-Don Delta shapefile (Appendix 3) are based on the data in this table and table A2.4 above.

| Country | ID | Sub-area | PC Cardiidae 20^th^ C | PC Hydrobiidae 20^th^ C | PC Cardiidae 21^st^ C | PC Hydrobiidae 21^st^ C | Remarks |
| --- | --- | --- | --- | --- | --- | --- | --- |
| RU | 5 | Coastal limans Kuban delta incl. Akhtarsk Liman | + Mordukhay-Boltovskoy (1960) | + Golikov and Starobogatov (1972) | + Korpakova et al. (2008), Korpakova et al. (2007) | Data deficient | *Dreissena* community present (Korpakova et al., 2010) |

**
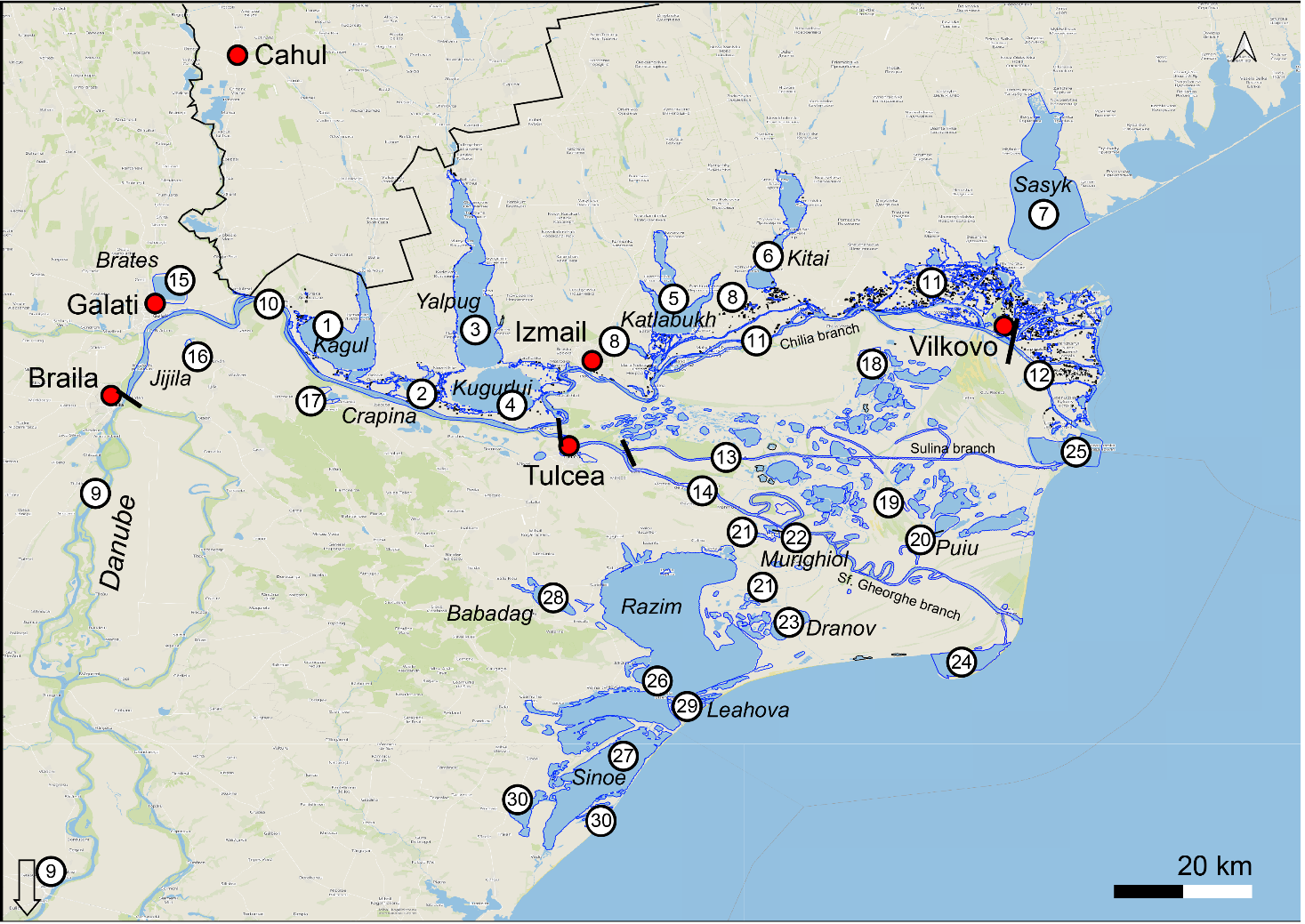
**

Fig. A2.1. Danube-Razim and Bulgarian coastal wetlands. See IDs of the sub-areas in Table A2.1. Map is projected in EPSG Projection 4326 - WGS 84.

**
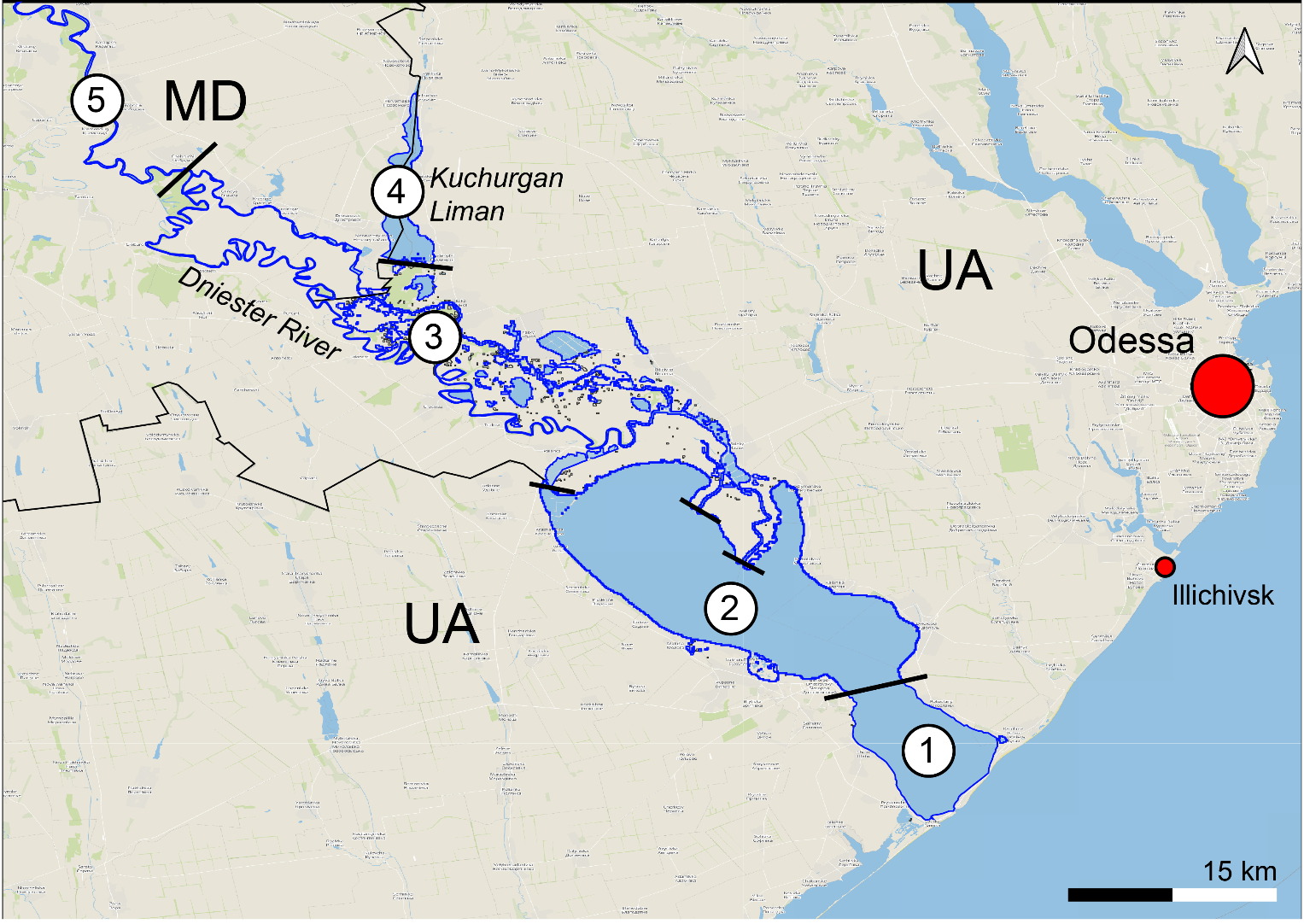
**

Fig. A2.2. Dniester Liman. See IDs of the sub-areas in Table A2.1. Map is projected in EPSG Projection 4326 - WGS 84.

**
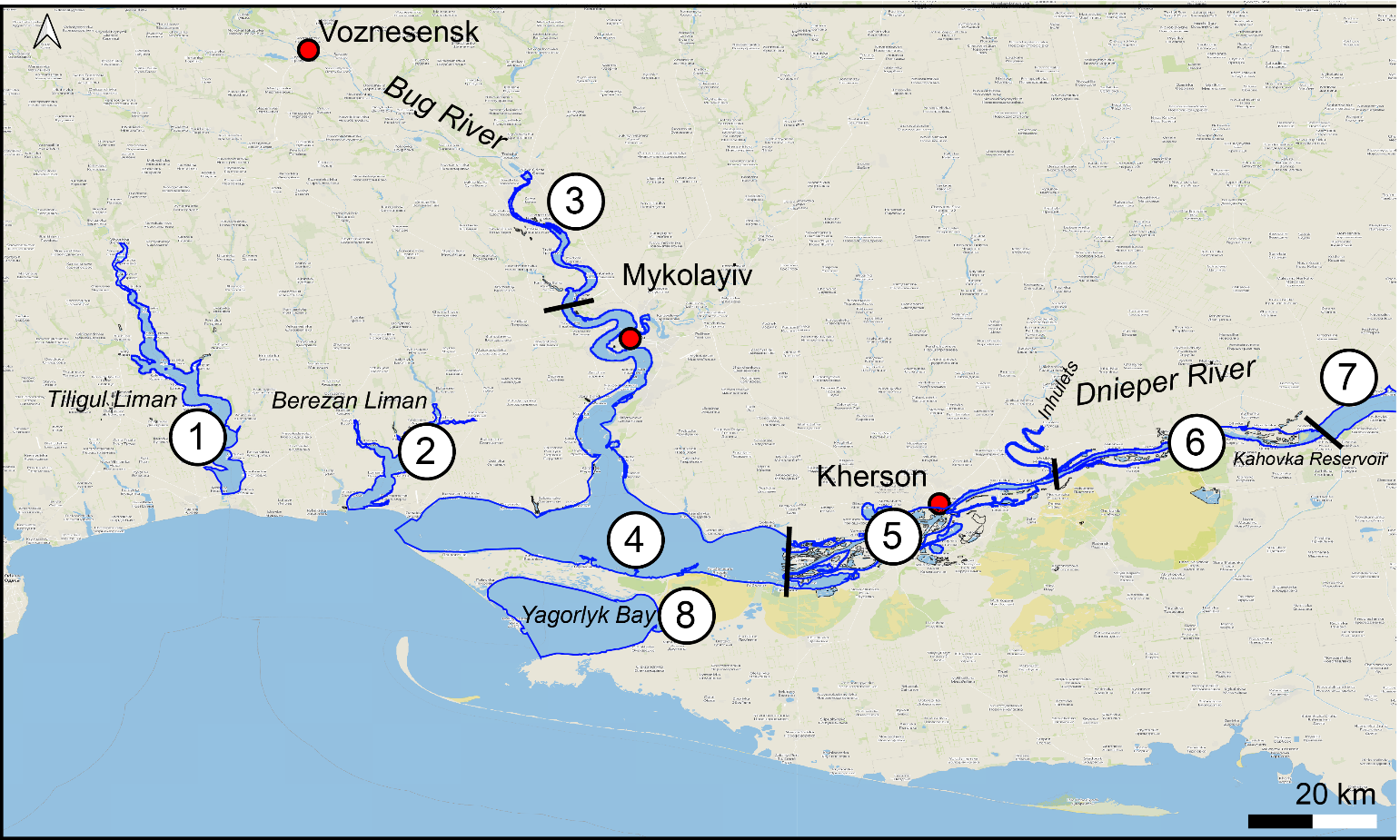
**

Fig. A2.3. Dnieper-Bug Estuary. See IDs of the sub-areas in Table A2.1. Map is projected in EPSG Projection 4326 - WGS 84.


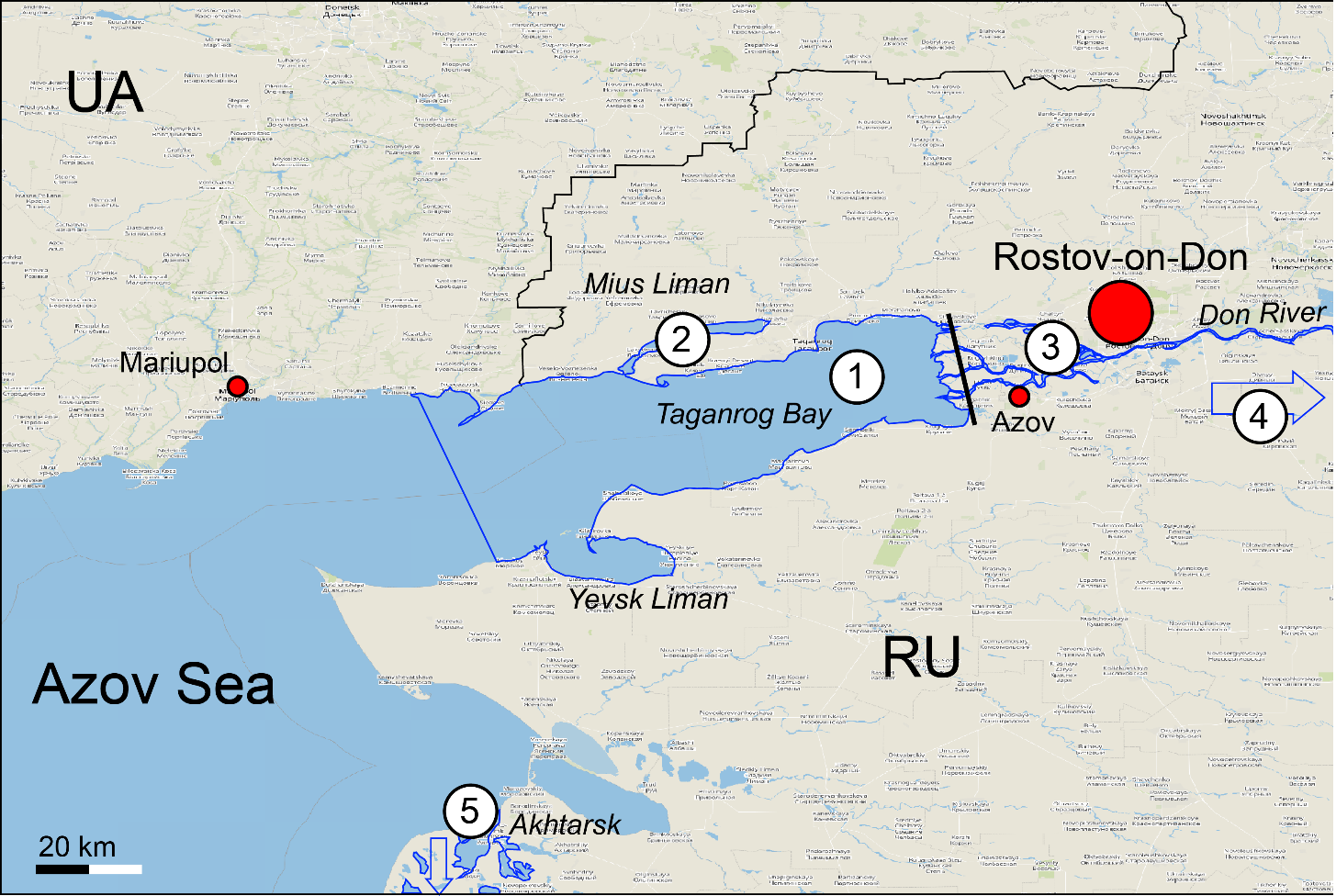


Fig. A2.4. Taganrog Bay-Don Delta and SE Azov Sea coast. See IDs of the sub-areas in Table A2.1. Map is projected in EPSG Projection 4326 - WGS 84.

**References**

Alexenko, T. L. 2004. Mollusks of the Dnieper-Bug Estuary Region and Their Role in Feeding of Fish. *Hydrobiological Journal,* 40**,** 56-62.

Angelov, A. 2000. Mollusca (Gastropoda et Bivalvia) aquae dulcis, catalogus Faunae Bulgaicae. *Pensoft & Backhuys Publ., Sofia, Leiden,* 54.

Anistratenko, V. V. 1996. Bryukhonogiye mollyuski Chernomorskogo biosphernogo zapovednika [Gastropod Mollusks of the Black Sea Biosphere Nature Reserve]. *Vestnik zoologii,* 1/2**,** 9-15.

Anistratenko, V. V. 2007. Finding of the extremely rare hydrobiid *Caspia logvinenkoi* (Mollusca: Gastropoda) in the estuary of the River Don and its zoogeographical significance. *Mollusca,* 25**,** 23-26.

Balashov, I. A., Son, M. O., Coadă, V. & Welter-Schultes, F. 2013. An updated annotated checklist of the molluscs of the Republic of Moldova. *Folia Malacologica,* 21**,** 175-181.

Boeters, H. D., Glöer, P., Georgiev, D. & Dedov, I. 2015. A new species of *Caspia* Clessin et W. Dybowski, 1887 (Gastropoda: Truncatelloidea: Hydrobiidae) in the Danube of Bulgaria. *Folia Malacologica,* 23**,** 177-186.

Bulysheva, N. I., Glushchenko, G. Y., Kreneva, K. V., Kleschenkov, A. V. & Varchenko, E. A. 2019. Settling of the fouling organisms at the metals in the delta of the Don River and in the estuarine zone of the Sea of Azov in winter. *International Multidisciplinary Scientific GeoConference: SGEM,* 19**,** 521-526.

Cartea Roșie a Republicii Moldova 2015. Chișinău: Știința.

Drensky, P. 1947. Synopsis and distribution of freshwater Mollusca in Bulgaria. *Godishnik na Sofiyskiya Ouniversitet, FMF, Kniga,* 43**,** 33-51. [in Bulgarian, English summary].

Dzhurtubaev, Y. M., Dzhurtubaev, М. & Zamorov, V. 2018. Macrozoobenthos of Danubian Lake Kugurluy (Odessa region, Ukraine). *Ukrainian Journal of Ecology,* 8**,** 898-905. [in Russian].

Dzhurtubaev, Y. M., Dzhutubaev, M. & Zamorov, V. 2017. Macrozoobenthos of Danubian Lake Yalpug (Odessa region, Ukraine). *Ukrainian Journal of Ecology,* 7**,** 160-168. [in Russian].

Filipenko, S. 2011. The current state of the benthic communities of the Kuchurgan Reservoir – the cooler of the Moldavian GRES // Vestnik Pridnestrovskogo Universiteta. *SER.: Mediko-Biologicheskie i Himicheskie Nauki,* 2**,** 83-90.

Golikov, A. N. & Starobogatov, Y. I. 1972. Klass Bryukhonogiye Mollyuski – Gastropoda Cuvier, 1797 (Mollusca-Gastropoda). *Opredelitel' Fauny Chernogo i Azovskogo Morey [Identification key to the fauna of the Black and Azov Seas, free living invertebrates: Arthropoda (besides Crustacea), Mollusca, Echinodermata, Chaetognatha, Chordata],* 3**,** 65-166. [in Russian].

Gomoiu, M. & Skolka, M. 1996. Changements recents dans la biodiversite de la Mer Noire dus aux immigrants. *Geo-eco-marina,* 1**,** 34-48.

Gomoiu, M. & Skolka, M. 1998. Cresterea biodiversitatii prin imigrare-noi specii in fauna Romaniei. *Increase of biodiversity by immigration-new species for the Romanian fauna. “Ovidius” University Annals of Natural Science, Biology-Ecology series,* 2**,** 181-202.

Grinbart, S. B. 1953a. Bentos Dnestrovskogo limana i nizoviev Dnestra, ego kormovaya otsenka // Materiali Materialy po gidrobiologii i rybolovstvu limanov severo-zapadnogo Prichernomoria [Benthos of the Dniester estuary and the lower reaches of the Dniester, assessment of its nutritional value // Materials on hydrobiology and fishing in the limans of the northwestern Black Sea]. *Odessa University***,** 7-17.

Grinbart, S. B. 1953b. K izucheniyu zoobentosa Tiligulskogo limana i ego kormovykh resursov [On the study of zoobenthos of the Tiligul estuary, and its feeding resources]. *Odessa University***,** 85-106. [in Russian].

Grossu, A. V. 1986. *Gastropoda Romaniae*, Editura Litera.

Hubenov, Z. 2015. Species composition of the free living multicellular invertebrate animals (Metazoa: Invertebrata) from the Bulgarian sector of the Black Sea and the coastal brackish basins. *Historia naturalis bulgarica,* 21**,** 49-168.

ICPDR 2008. Joint Danube Survey 2, Final Scientific Report. *ICPDR Secretariat, Vienna International Centre.* Vienna, Austria.

ICPDR 2015. Joint Danube Survey 3. A comprehensive analysis of Danube water quality. Vienna.

Ignat, G., Cristofor, S., Angheluță, V., Rîșnoveanu, G., Nafornița, G. & Florescu, C. 1997. Structure and dynamics of benthic fauna in Danube Danube and Danube Delta, Scientific Annals of the Danube Delta Research and Design Institute. IV**,** 133-142.

Kaneva-Abadjieva, V. 1957. Mollusca and Malacostraca im Varnasee. *Arbeiten aus der Biologischen Meeresstation, Varna,* 19**,** 127–154. [in Bulgarian, Russian and German summaries].

Khalaim, А. А. & Son, М. О. 2016. Biologo-ekologicheskaya kharakteristika *Hypanis laeviuscula fragilis* (Milachevitch, 1908)(Mollusca, Cardiidae) vodokhranilishcha Sasyk [Biological and ecological characteristics of *Hypanis laeviuscula fragilis* (Milachevitch, 1908)(Mollusca, Cardiidae) from the Sasyk reservoir]. *Uzhgorod University Scientific Bulletin: Series: Biology***,** 59-63. [in Russian].

Korpakova, I., Afanasyev, D., Barabashin, T., Tsybulsky, I., Belova, L., Naletova, L. & Bychkova, M. 2007. Gidrobiologicheskiye osobennosti limanno-plavnevoy zony Temryukskogo zaliva Azovskogo morya [Hydrobiological features of the estuary area of the Temryuk Bay of the Sea of Azov]. *Environmental Protection in the Oil and Gas Complex,* 9**,** 69-75. [in Russian].

Korpakova, I., Afanasyev, D., Tsybulsky, I., Barabashin, T., Belova, L., Naletova, L. & Bychkova, M. 2010. Bentosnyye i planktonnyye soobshestva limanov i plaven del'ty reki Kuban' [Benthic and planktonic communities of estuaries of the Kuban River delta]. *News of higher educational institutions. North Caucasus region. Natural sciences,* 2**,** 78-81. [in Russian].

Korpakova, I., Tsybulsky, I., Afanasyev, D., Barabashin, T., Belova, L., Naletova, L. & Bychkova, M. 2008. Gidrobiologicheskiye osobennosti yugo-vostochnoy chasti Azovskogo morya [Hydrobiological characteristics of the south-eastern part of the Sea of Azov]. *Environmental Protection in the Oil and Gas Complex,* 11**,** 70-80. [in Russian].

Kovachev, S., Stoichev, S. & Hainadjieva, V. 1999. The zoobenthos of several lakes along the Northern Bulgarian Black Sea Coast. *Lauterbornia,* 35**,** 33-38.

Kovalenko, E. P. Donnyye bespozvonochnyye krupnykh vodotokov del'ty Dona (Bottom invertebrates of large watercourses of the Don delta). VI International Scientific and Practical Conference of Young Scientists, dedicated to the ecological problems of aquatic ecosystems, 2009 Sevastopol. 55. [in Russian].

Markovsky, Y. M. 1953. Fauna bespozvonochnykh nizov'ev rek Ukrainy, usloviya sushchestvo vaniya i puti ispol'zovaniya. Chast' 1. Vodoemy delty Dnestra i Dnestrovskiy liman [Fauna of invertebrates of the lower river streams of Ukraine, life conditions and ways of utilization. Part 1. The basin of the Dniester delta and Dniester lagoon]. AN USSR, Kiev**,** 1-207. [in Russian].

Markovsky, Y. M. 1954. Fauna bespozvonochnykh nizov'ev rek Ukrainy, usloviya sushchestvovaniya i puti ispol'zovaniya. Chast' 2. Dneprovsko-Bugskiy liman [Fauna of invertebrates of the lower river streams of Ukraine, life conditions and ways of utilization. Part 2. Dnieper-Bug lagoon]. *AN USSR, Kiev***,** 1-207. [in Russian].

Markovsky, Y. M. 1955. Fauna bespozvonochnykh nizov'ev rek Ukrainy, usloviya sushchestvovaniya i puti ispol'zovaniya. Chast' 3. Vodoemy Kilijskoy delty Dunaya [The fauna of invertebrates of the lower river streams of Ukraine, life conditions and ways of utilization. Part 3. The basin of the Kilian delta of the Danube]. *AN USSR, Kiev***,** 1-275. [in Russian].

Mihailova-Neikova, M. 1961. Hydrobiological research of the Mandra Lake with regard to its importance as a fishing ground. *Godishnik na Sofiyskiya Ouniversitet, BGGF, Kniga,* 53 (1. Zoology)**,** 57-123. [in Bulgarian].

Mordukhay-Boltovskoy, F. 1960. Kaspiyskaya fauna v Azovo-Chernomorskom basseyne. *Izdatel’stvo Akademii Nauk SSSR, Leningrad***,** 228 pp. [in Russian].

Munasypova-Motyash, I. A. 2006. O sovremennoy faune dvustvorchatykh mollyuskov podsemeistva Limnocardiinae (Bivalvia, Cardiidae) Severo-Zapadnogo Prichernomorya [On the recent fauna of subfamily Limnocardiinae (Bivalvia, Cardiidae) in North-Western shore of Black Sea]. *Vestnik zoologii,* 40**,** 41-48. [in Russian].

Munjiu, O. The modern species composition of freshwater mollusks from Moldova. Geoecological and bioecological problems of the north Black Sea coast, 2012 Tiraspol. 205–207. [in Russian].

Nabozhenko, M. V. 2005. Raspredeleniye dvustvorchatykh mollyuskov roda *Hypanis* Pander in Ménétriés, 1832 (Bivalvia, Cardioidea: Limnocardiidae) v Taganrogskom zalive (Azovskoye more) [Distribution of the genus *Hypanis* Pander in Ménétriés, 1832 (Bivalvia, Cardioidea: Limnocardiidae) in the Taganrog Gulf (The Sea of Azov)]. *Ekologiya Morya***,** 44.

Nabozhenko, M. V. 2008. Rasprostraniyeniye mollyuskov podsemeystva Lymnocardiinae (Bivalvia, Cardiidae) v basseyne Azovskogo morya [Distribution of Mollusks of the Subfamily Lymnocardiinae (Bivalvia, Cardiidae) in the Basin of the Azov Sea]. *Vestnik. YuNTs RAN,* 4**,** 78-82. [in Russian].

Nekrasova, M. Y. 1972. Zoobentos Azovskogo morya posle zaregulirovaniya stoka Dona [Zoobenthos of the Azov Sea after the control of the Don River]. *Zoologicheskii Zhurnal,* 51**,** 789-798. [in Russian].

Pandourski, I. 2001. Recherches hydrobiologiques des zones humides de la côte bulgare de la Mer Noire. I. Le lac de Vaja. *Riv. Idrobiol,* 40**,** 321-334.

Paraschiv, G.-M., Begun, T., Teaca, A., Bucur, M. & Tofan, L. 2010a. New data about benthal populations of the Golovita and Zmeica lakes. *Journal of Environmental Protection and Ecology,* 11**,** 253-260.

Paraschiv, G.-M., Tofan, L., Schroder, V. & Bucur, M. 2010b. Analysis of zoobenthal communities from the Razim-sinoe lagoon complex. *Journal of Environmental Protection and Ecology,* 11**,** 261-268.

Pavel, A. B., Menabit, S., Mânzală, D., Lupașcu, N., Pop, I. C. & Catianis, I. 2019. Benthic community structure characterization of the bed-sediment layer composition in the Musura Bay and Sakhalin area. *Geo-Eco-Marina,* 25**,** 15-29.

Popa, O. P., Sarkany-Kiss, A., Kelemen, B. S., Iorgu, E. I., Murariu, D. & Popa, L. O. 2009. Contributions to the knowledge of the present Limnocardiidae fauna (Mollusca: Bivalvia) from Romania. *Travaux du Muséum National d’Histoire Naturelle ‘‘Grigore Antipa,* 52**,** 7-15.

Russev, B. K. 1966. The zoobenthos of Danube River between 845th and 375th river kilometer. *I. Diversity, distribution and ecology. Izv. Zool. Inst. Muz. Sofia,* 20**,** 55-131.

Scarlato, O. A. & Starobogatov, Y. I. 1972. Klass Dvustvorchatye Mollyuski – Bivalvia Linné, 1758 [Mollusca-Bivalvia]. *Opredelitel' Fauny Chernogo i Azovskogo Morey [Identification key to the fauna of the Black and Azov Seas, free living invertebrates: Arthropoda (besides Crustacea), Mollusca, Echinodermata, Chaetognatha, Chordata],* 3**,** 178-270. [in Russian].

Semenchenko, V., Son, M., Novitsky, R. A., Kvach, Y. & Panov, V. E. 2016. Checklist of non-native benthic macroinvertebrates and fish in the Dnieper River basin. *BioInvasions Records,* 5**,** 185–187.

Shokhin, I. V., Nabozhenko, M. V., Sarvilina, S. V. & Titova, E. P. 2006. The present-day condition and regularities of the distribution of the bottom communities in Taganrog Bay. *Oceanology,* 46**,** 401-410.

Son, M. O. 2007. Invasive molluscs in fresh and brackish waters of the Northern Black Sea Region. *Druk, Odessa***,** 1-131.

Son, M. O., Prokin, A. A., Dubov, P. G., Konopacka, A., Grabowski, M., MacNeil, C. & Panov, V. E. 2020. Caspian invaders vs. Ponto-Caspian locals – range expansion of invasive macroinvertebrates from the Volga Basin results in high biological pollution of the Lower Don River. *Management of Biological Invasions,* 11**,** 178-200.

Stark, I. 1960. Bentos Taganrogskogo zaliva. [Benthos of the Taganrog Bay] *Trudy Azovskogo Nauchno-Issledovatel'skogo Instituta Rybnogo Khozyaystva (AZNIIRKH),* 1**,** 210-216. [in Russian].

Stoica, C., Gheorghe, S., Petre, J., Lucaciu, I. & Nita-Lazar, M. 2014. Tools for assessing Danube Delta systems with macro invertebrates. *Environmental Engineering & Management Journal (EEMJ),* 13.

Stoica, C., Lucaciu, I., Nicolau, M. & Vosniakos, F. 2012. Monitoring the ecological diversity of the aquatic Danube Delta systems in terms of spatial-temporal relationship.

Stoica, C., Stanescu, E., Lucaciu, I., Gheorghe, S. & Nicolau, M. 2013. Influence of global change on biological assemblages in the Danube Delta.

Teodorescu-Leonte, R. 1966. Rezultatele Cercetărilor asupra bazei trofice a complexului Razelm şi perspectivele producţiei piscicole din acest complex prin dirijarea popularii. *Bul. Inst. Cerc. Project. Piscicole,* 25**,** 38-46.

Teodorescu-Leonte, R. 1977. Le complexe Razelm–Sinoie un sistem typiquement saumatre. MAMBO Constanta. *In „Biologie des eauxsaumatre de la Mer Noire”***,** 213-234.

Teodorescu-Leonte, R. & Leonte, V. 1969. Variation des biocenoses benthiques du Complexe Razelm, en fonction des conditions de salinité. *Rapports de la Commission Internationale pour l'Étude Scientifique de la Mer Méditerranée. CIESM, Monaco 19 (5).*

Teodorescu-Leonte, R., Leonte, V., Dumitru, M. & Soileanu, B. 1956. Observations on the Razelm–Sinoie complex during 1950–1952. *The Annals of the Romanian Research Institute of Fisheries,* 1**,** 1-50.

Vǎdineanu, A., Cristofor, S., Ignat, G., Ciubuc, C., Rîşnoveanu, G., Bodescu, F. & Botnariuc, N. 2000. Structural and functional changes within the benthic communities of Danube Delta lakes. *Internationale Vereinigung für theoretische und angewandte Limnologie: Verhandlungen,* 27**,** 2571-2576.

Valkanov, A. 1941. Our coastal lakes and marshes in Dobrudzha. *Fishery Review [Ribarski Pregled],* 6. [in Bulgarian].

Valkanov, A. 1957. Katalog unserer Schwarzmeerfauna. *Arbeiten aus der Biologischen Meeresstation in Varna,* 19**,** 1–62. [in Bulgarian].

Velde, S. v. d., Jorissen, E. L., Neubauer, T. A., Radan, S., Pavel, A. B., Stoica, M., Van Baak, C. G., Martínez Gándara, A., Popa, L. & Stigter, H. d. 2019. A conservation palaeobiological approach to assess faunal response of threatened biota under natural and anthropogenic environmental change. *Biogeosciences,* 16**,** 2423-2442.

Vidinova, Y., Tyufekchieva, V., Varadinova, E., Stoichev, S., Kenderov, L., Dedov, I. & Uzunov, Y. 2016. Taxonomic list of benthic macroinvertebrate communities of inland standing water bodies in Bulgaria. *Acta Zoologica Bulgarica,* 68**,** 147-158.

Vorobyev, V. P. 1949. Benthos Azovskogo morya [Benthos of the Sea of Azov]. *Proceedings of the Azov-Black Sea Institute of Marine Fisheries and Oceanography,* 13**,** 1-196.

Wilke, T., Albrecht, C., Anistratenko, V. V., Şahin, S. K. & Yildirim, M. Z. 2007. Testing biogeographical hypotheses in space and time: faunal relationships of the putative ancient Lake Eǧirdir in Asia Minor. *Journal of Biogeography,* 34**,** 1807-1821.

Wohlberedt, O. 1911. Zur Molluskenfauna von Bulgarien. *Abhandlungen der Naturforschenden Gesellschaft zu Gorlitz,* 27**,** 167-234.

Zhadin, V. 1931. Die Mollusken des Bassins des südlichen Bugs. *Trudy Prirodnico-Technicnogo Viddilu,* 13**,** 13–53.

Zhivoglyadova, L. A. & Frolenko, L. N. 2017. Kharakteristika kormovoi bazi rib-bentofagov nijnego Dona [Characteristics of the food supply for benthophagous fish in the Lower Don]. *Izvestiya TINRO (Transactions of the Pacific Research Institute of Fisheries and Oceanography),* 189. [in Russian].
